# Incidence and risk factors for stillbirth in Kenya, Mozambique and the Gambia: the PRECISE cohort study

**DOI:** 10.1016/j.lanafr.2026.100089

**Published:** 2026-08

**Authors:** Grace Mwashigadi, Joseph Akuze, Angela Koech, Hawanatu Jah, Anna Roca, Umberto D'Alessandro, Esperança Sevene, Sonia Maculuve, Anifa Valá, Sanjukta Singh, Bethany Atkins, Geoffrey Omuse, Moses Mukhanya, Marie-Laure Volvert, Laura Magee, Rachel Craik, Marleen Temmerman, Peter von Dadelszen, Hannah Blencowe, the PRECISE Network, Umberto D’Alessandro, Anna Roca, Hawanatu Jah, Andrew Prentice, Melisa Martinez-Alvarez, Brahima Diallo, Abdul Sesay, Sambou Suso, Baboucarr Njie, Fatima Touray, Yahaya Idris, Fatoumata Kongira, Modou F.S. Ndure, Lawrence Gibba, Abdoulie Bah, Yorro Bah, Marleen Temmerman, Amina Abubakar, Akbar K. Waljee, Angela Koech, Patricia Okiro, Geoffrey Omuse, Grace Mwashigadi, Consolata Juma, Joseph Mutunga, Moses Mukhanya, Onesmus Wanje, Isaac Mwaniki, Marvin Ochieng, Emily Mwadime, Esperança Sevene, Corssino Tchavana, Salesio Macuacua, Anifa Vala, Helena Boene, Lazaro Quimice, Sonia Maculuve, Inacio Mandomando, Peter von Dadelszen, Laura A. Magee, Rachel Craik, Marie-Laure Volvert, Hiten Mistry, Thomas Mendy, Donna Russell, Prestige Tatenda Makanga, Liberty Makacha, Reason Mlambo, Lucilla Poston, Rachel Tribe, Sophie Moore, Tatiana Taylor Salisbury, Aris Papageorghiou, Alison Noble, Rachel Craik, Hannah Blencowe, Veronique Filippi, Joy Lawn, Matt Silver, Joseph Akuze, Ursula Gazeley, Judith Cartwright, Guy Whitley, Sanjeev Krishna, Marianne Vidler, Jing (Larry) Li, Jeff Bone, Mai-Lei (Maggie) Woo Kinshella, Domena Tu, Ash Sandhu, Kelly Pickerill, Carla Carillho, Benjamin Barratt

**Affiliations:** a Centre of Excellence in Women and Child Health, Aga Khan University, Nairobi, Kenya; b Centre for Maternal Reproductive Adolescent, and Child Health (MARCH), London School of Hygiene & Tropical Medicine, London, UK; c Department of Obstetrics and Gynaecology, Aga Khan University, Nairobi, Kenya; d Medical Research Council Unit, The Gambia at the London School of Hygiene, and Tropical Medicine, Fajara, The Gambia; e Department of Obstetrics & Gynaecology, Amsterdam University Medical Center Location University of Amsterdam, Amsterdam, the Netherlands; f ISGlobal, Barcelona Institute for Global Health, Campus Hospital Clínic-Universitat de Barcelona, Barcelona 08036, Spain; g ICREA, Pg. Lluís Companys 23, Barcelona 08010, Spain; h Centro de Investigação em Saúde de Manhiça, Manhiça, Mozambique; i Department of Physiological Science, Clinical Pharmacology, Faculty of Medicine, Eduardo Mondlane University, Mozambique; j Institute for Women's Health, University College London, London, UK; k Department of Pathology, Aga Khan University, Nairobi, Kenya; l Department of Women and Children’s Health, School of Life Course and Population Sciences, Kings College London, London, UK; m University of Oxford, Oxford, UK

**Keywords:** Stillbirth, Rates, Risk factors, sub-Saharan Africa

## Abstract

**Background:**

Stillbirth is a critical public health challenge in sub-Saharan Africa, but prospective data on incidence and risk factors remain limited. We conducted a multi-country study in Kenya, Mozambique, and The Gambia to determine stillbirth rates and underlying risk factors.

**Methods:**

We analysed pooled data from 5772 women aged 16–49 with birth outcomes enrolled from seven health facilities in Mozambique, Kenya, and The Gambia participating in the PRECISE (PREgnancy Care Integrating translational Science, Everywhere) cohort (2019–2022). We used bivariable and multivariable modified Poisson regression models to assess associations between maternal socio-demographic, environmental, medical, including obstetric, and health system factors with stillbirth (≥20 weeks gestation or weighing ≥500 g).

**Findings:**

Overall stillbirth incidence was 29.2 per 1000 total births (95%CI 24.8–33.6): 17.5 (95% CI:12.5–22.5) in Kenya, 32.8 (95% CI:24.7–40.9) in Mozambique, and 49.8 (95%CI: 37.4–62.4) in The Gambia. The majority (89.1%) occurred at ≥28 weeks gestation. Significant risk factors were a history of previous stillbirth (attributable risk ratio (aRR) = 3.18, 95% CI: 1.85–5.46), Stage 2 hypertension (aRR = 3.25, 95% CI: 1.42–7.44), antepartum haemorrhage (aRR = 3.10, 95% CI: 1.77–5.42), Caesarean delivery aRR = 1.74 (95%CI:1.01–3.01), and small-for-gestational age (aRR = 2.05 (95%CI:1.34–3.12)).

**Interpretation:**

Stillbirth rates remain unacceptably high across these settings, with country variations. Multifactorial determinants underscore the need for integrated antenatal and intrapartum care strategies addressing systemic barriers to early detection of risk, robust referral systems and timely access to high-quality emergency obstetric care towards ending preventable stillbirths in these contexts.

**Funding:**

The PRECISE Network was funded by the UK Research and Innovation Grand Challenges Research Fund GROW Award scheme (grant number: MR/P027938/1). The PRECISE cohort extension in Kenya after January 2022 was funded by the 10.13039/100000179Office of the Director, 10.13039/100000002National Institutes of Health, the 10.13039/100000070National Institute of Biomedical Imaging and Bioengineering, the 10.13039/100000025National Institute of Mental Health, and the 10.13039/100000061Fogarty International Center AT THE NATIONAL INSTITUTES OF HEALTH under award number U54TW012089. The funders had no role in study design, data collection and analysis, decision to publish, or preparation of the manuscript.

**Research in context**

**Evidence before this study**

We searched PubMed and Embase for studies of stillbirth incidence and risk factors in sub-Saharan Africa published between 1 January 2000 and 31 December 2024, using combinations of the terms “stillbirth”, “perinatal death”, “foetal death”, “Africa”, “sub-Saharan” and individual country names, with no language restriction. Reference lists of identified reviews and UN Inter-agency Group for Child Mortality Estimation reports were hand-searched. Existing evidence indicated that sub-Saharan Africa carried the highest global stillbirth burden, with rates more than 7-fold higher than high-income countries. Recurring risk factors included hypertensive disorders, antepartum haemorrhage, infections and inadequate skilled deliveries, alongside socio-demographic determinants (low maternal education, poverty, advanced age, grand multiparity). However, most previous studies were single-site facility-based, recruited at the time of birth, relied on retrospective data collection, gestational age assessment at time of birth, and excluded early stillbirths (20–27 weeks). Prospective cohort data with early gestational age assessment and systematic risk factor capture from across multiple African settings remained scarce.

**Added value of this study**

This prospective multi-country cohort recruited 5772 women at first antenatal care attendance across Kenya, Mozambique and The Gambia, with systematic best-obstetric-estimate gestational age assessment and capture of all stillbirths from 20 weeks. The overall stillbirth rate was 29.2 per 1000 total births, with substantial between-country variation (17.5 in Kenya to 49.8 in The Gambia), and 10.9% of stillbirths occurred at 20–27 weeks; losses that would be invisible under the standard ≥28-week threshold. A hierarchical analysis spanning distal, intermediate and proximal determinants identified previous stillbirth, Stage 2 hypertension, antepartum haemorrhage, small-for-gestational age, and Caesarean delivery as independent risk factors, and quantified the contribution of small-for-gestational age, a measure inadequately characterised in previous African studies.

**Implications of all the available evidence**

Stillbirths remain unacceptably high in sub-Saharan Africa and reducing them requires integrated approaches addressing clinical and health-system factors simultaneously. Better management of hypertensive disorders and timely emergency obstetric care for antepartum haemorrhage have the potential to substantially reduce stillbirths. The persistent association of caesarean section with stillbirth risk likely signals systemic weaknesses in intrapartum monitoring and emergency response. The threefold risk associated with previous stillbirth highlights the needs for enhanced post-stillbirth care, bereavement support and individualised subsequent pregnancy management and surveillance. Early stillbirths (20–27 weeks) represent a previously underappreciated burden that requiring early antenatal care engagement.

Future work should develop and validate antenatal risk-stratification tools combining maternal history, clinical markers and biomarkers, and translate findings into implementation-ready interventions to accelerate progress toward the Every Newborn Action Plan targets.

## Introduction

Stillbirth is a devastating pregnancy outcome that causes profound emotional distress for parents, families and health providers, and remains a significant global concern^1^^,^^2^ In 2023, approximately 1.9 million pregnancies worldwide ended in late gestation stillbirth, at ≥28 weeks gestation.^3^ Most (98%) of these deaths were in low- and middle-income countries (LMICs), with more than 60% of them in rural regions, impacting disproportionally families with lower socioeconomic status and limited access to maternal healthcare.^4^, ^5^, ^6^ The majority of cases worldwide could be prevented through improved maternal healthcare.^4^^,^^7^^,^^8^ While global commitments to prioritize preventive efforts have been made, progress for many LMICs is insufficient to reach the Every Newborn Action Plan (ENAP) target of each country achieving a late gestation stillbirth rate of 12 or fewer per 1000 total births by 2030.^3^

Importantly, sub-Saharan Africa’s contribution to the global stillbirth burden is increasing. In 2000, 26% of late gestation stillbirths worldwide occurred in Africa, increasing to 48% by 2023.^3^ Whilst over the last two decades some decline in stillbirth rates in Africa has been seen, this has been modest compared to the progress in other high-burden regions such as Southern Asia.^3^ Despite the substantial under-reporting due to poor data systems and the reliance on modelling,^3^ the estimated overall stillbirth rate in sub-Saharan Africa in 2023 was ten-fold higher (22.2 per 1000 total births) than in Europe, Northern America, and Australia (2.8 per 1000 total births).^3^ The stillbirth rates in Kenya, The Gambia and Mozambique are 22.5, 27.1 and 27.8 per 1000 total births respectively, according to United Nations (UN) global estimates. These UN estimates currently include only stillbirths at ≥28 weeks’ gestation, leaving stillbirths occurring from 20^+0^ weeks to 27^+6^ weeks’ gestation uncounted.^3^

Whilst previous African studies have found stillbirth to be associated with maternal, obstetric, socio-economic, and health service-related factors, these have been largely retrospective hospital-based studies lacking generalisability at the population level, frequently including only^5^^,^^6^^,^^9^^,^^10^ late gestation stillbirths. In addition, the implementation of timely and accurate gestational age assessment via early ultrasonography, critical for differentiating stillbirths from previable fetal losses, remains inconsistent in both routine clinical practice and research settings.^11^ Improved understanding of risk factors for all stillbirths (≥20 weeks), including potentially modifiable factors across different African contexts, is needed to target interventions to prevent these deaths. There is an urgent public health imperative to close these data gaps and generate contemporary, context-specific evidence on the incidence and modifiable risk factors for stillbirth to inform investment and policies to end this silent crisis.

This study aims to address these gaps by describing the incidence of all stillbirths and associated risk factors across the PRECISE network’s prospective cohort three sites in Africa: Kenya, Mozambique, and The Gambia.

## Methods

### Study setting and design

The PRECISE (PREgnancy Care Integrating translational Science, Everywhere) was a prospective observational cohort established to investigate specific placental disorders (pregnancy hypertension, preterm birth, fetal growth restriction, and stillbirth) in sub-Saharan Africa. The study protocol and cohort description have been published previously.^12^^,^^13^ This study is reported in accordance with the STROBE (Strengthening the Reporting of Observational Studies in Epidemiology) guidelines (Supporting information S1).

### Study population

Between June 2019 and December 2022, pregnant women aged 16–49 years were recruited from government/public health study facilities in Kenya: Mariakani Subcounty Hospital (urban population) and Rabai Subcounty Hospital (primarily rural community), The Gambia: Farafenni Hospital (urban hospital) and Illiasa, and Ngayen Sanjal (rural); Mozambique: Manhiça District Hospital (urban) and Xinavane Rural Hospital (rural). Inclusion of adolescents in PRECISE was informed by the high prevalence of teenage pregnancies and was approved by all site ethics committees. Women were enrolled into the ‘unselected pregnancy cohort’ at the first antenatal care (ANC) visit and if they planned to deliver at that facility. Recruitment followed a consecutive sampling approach, whereby all eligible women presenting for their first ANC visit at participating facilities during the enrolment period were invited to participate. Women who were referred to that facility were not eligible. For the ‘time of disease’ cohort, women were enrolled at the time a diagnosis of hypertension, suspected FGR, intrauterine fetal death (IUFD) or stillbirth was made - during ANC, during admission for delivery or immediately after birth. The sample size for this analysis was determined by the parent PRECISE study, the design and recruitment targets of which have been described previously.^12^^,^^13^ The present analysis includes all women with recorded birth outcomes.

### Data collection and management

Baseline demographic, clinical, and non-clinical data, and biological samples, were collected during recruitment and follow-up visits from pregnancy through 6 weeks postpartum.

Data were collected by trained research assistants (research nurses and data entry clerks) fluent in both the language of the questionnaire and the common local languages. Data were collected directly from women or from their healthcare records using standardized data collection forms and entered directly into the PRECISE, using electronic data capture database.^12^^,^^13^ The study questionnaire was developed from preexisting databases and standard tools, primarily from the COLLECT datasets and other maternal and child health measurement tools.^12^ Training and data collection were guided by standard operating procedures across all sites to ensure uniform understanding of each question and responses. In Mozambique, questionnaires were translated to Portuguese by members of the research and clinical teams prioritising conceptual and cultural equivalence over literal translation. Data were cleaned and managed using a standard protocol and stored on study-specific servers in each host institution, and back-ups of de-identified data stored on a remote server at the University of British Columbia’s Children's Hospital Research Institute.

Self-reported ethnicity was recorded at enrolment using site-specific category lists (>40 ethnic-group labels in total) reflecting populations served by recruiting facilities. Categories were not harmonised across sites, and within each site many groups contained few participants. Ethnicity therefore could not be analysed as a determinant of stillbirth in this cohort.

### Outcomes

**Stillbirth** was defined as delivery of a fetus with no signs of life at ≥20^+0^ weeks’ gestational age (GA) or weighing ≥500 g.^12^ This broad definition was chosen to capture all stillbirth cases. To facilitate comparison with other studies, we also report the prevalence using the WHO ICD-11 categories: ‘early gestation’ (22^+0^–27^+6^ weeks or 500–999 g if GA missing) and ‘late gestation’ (≥28^+0^ weeks or ≥1000 g if GA missing) using the definitions.^14^

GA was assessed using a best obstetric estimate hierarchy prioritising early ultrasound, if unavailable TraCer (a wireless ultrasound probe connected to an Android app enabling point of care videos to be later dated by skilled sonographers)^13^^,^^15^^,^^16^; then last menstrual period and symphysis-fundal measurement when other measures were absent (Supplementary information S2.1 and 2.2).

### Potential risk factors

A conceptual framework was developed guided by the hierarchical approach of Victora et al., which proposes that health determinants operate across hierarchical levels, with distal factors acting through intermediate and proximal pathways rather than having direct effects on the outcome.^17^ Potential risk factors, including maternal, fetal, obstetric, sociodemographic, and behavioural factors, were identified from the published literature.^6^^,^^9^^,^^10^^,^^17^, ^18^, ^19^, ^20^, ^21^ These risk factors were classified into proximal, intermediate, and distal categories based on their temporal relationship to stillbirth. Variables in **bold** were collected in PRECISE and explored in these analyses (Supplementary information S2.3 for further details). While preterm labour can lead to stillbirth, in many cases in-utero fetal death precedes preterm labour and resultant preterm birth is a consequence of the fetal death, rather than a contributor to it. To avoid this temporal ambiguity, GA at birth was excluded from our final multivariate analysis of stillbirth risk factors.

### Analyses

Stillbirth incidence rates were calculated overall and by country as the (number of stillbirths/total births) ∗1000 for each stillbirth definition, including women in the unselected pregnancy cohort only due to lack of denominator data in the time-of-disease cohort. All other analyses include women from both the unselected pregnancy and time-of-disease cohorts.

**Fig. 1:**
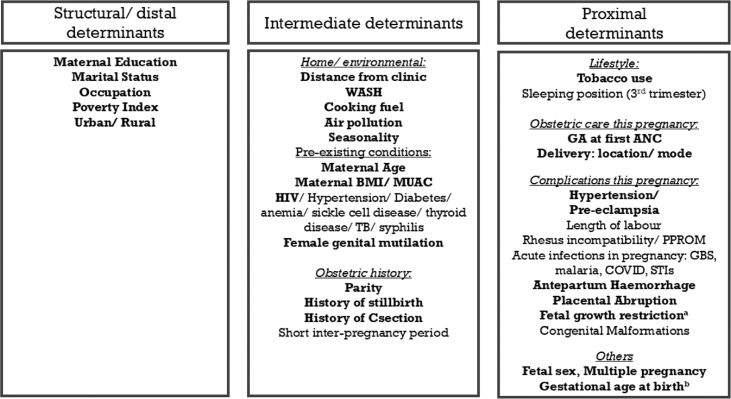
Conceptual framework of determinants of stillbirth (modified from Victora et al.). Framework modified from Victora et al.^1^ Factors in bold were available in the PRECISE dataset and considered in these analyses. BMI= Body Mass Index, MUAC = mid-upper arm circumference, TB = Tuberculosis, PPROM = pre-labour, premature rupture of membranes, GA = gestational age, ANC = Antenatal Clinic, GBS = Group B Streptococcus, STI = sexually transmitted infections. ^a^Small for gestational age (<10th Centile) at birth was used as a proxy. ^b^Gestational age at birth is strongly correlated with stillbirth outcome however many cases in-utero fetal death precedes preterm labour and resultant preterm birth is a consequence of the fetal death, rather than a contributor to it. To avoid this temporal ambiguity, GA at birth was excluded from our final multivariate analysis of stillbirth risk factors.

Data regarding potential stillbirth risk factors are presented by country using numbers (percentages) for categorical variables and median and interquartile range (IQR) for continuous variables. Potential risk factors were classified as distal, intermediate, or proximal based on the conceptual framework (Fig. 1).

We checked for multicollinearity between the independent variables and only included non-collinear variables with variance inflation factor (VIF) less than 10 and pairwise correlation coefficients below 0.5 in our analyses Supplementary information S4. Missing data for individual potential covariates was generally low (<5% overall). However, as missingness was differential and higher amongst stillbirths compared to livebirths, notably for size-for-gestational age 37.5% missing for stillbirths compared to 12.0% for livebirths complete case analyses was undertaken as the primary analysis. Sensitivity analysis was undertaken with missing data handled using multiple imputation by chained equations (MICE) with 25 imputed datasets (Supplementary information S5).

Bivariable modified Poisson regression was used to assess associations between risk factors and stillbirth outcomes. Factors associated with stillbirth on bivariable analysis (p < 0.2) without evidence of multi-collinearity (Variance Inflation Factor<10 and coefficient<0.5 on the correlation matrix) were added in order of magnitude to incremental multivariable Poisson regression models. Model 1: distal factors only, Model 2: distal and intermediate factors, and Model 3: distal, intermediate, and proximal factors. A forward approach was used, starting with the variable with the strongest evidence of association on bivariable analysis at each level. Akaike's information criterion (AIC) and Bayesian information criterion (BIC) were used to evaluate model fit and complexity for each sequential model, and variables retained where AIC and BIC improved. Associations are presented as adjusted risk ratios (aRRs) with 95% confidence intervals (CIs) derived from robust (sandwich) standard errors. Statistical significance was assessed using the Wald test at the alpha(α) = 0.05 level. All analyses were performed using Stata 18.

### Patient and public involvement and engagement

Community engagement was embedded throughout the PRECISE study, ensuring women and their communities shaped the research. Across all sites, 369 meetings involving 6866 participants and community members were conducted.

## Results

### Characteristics of participants

**Fig. 2:**
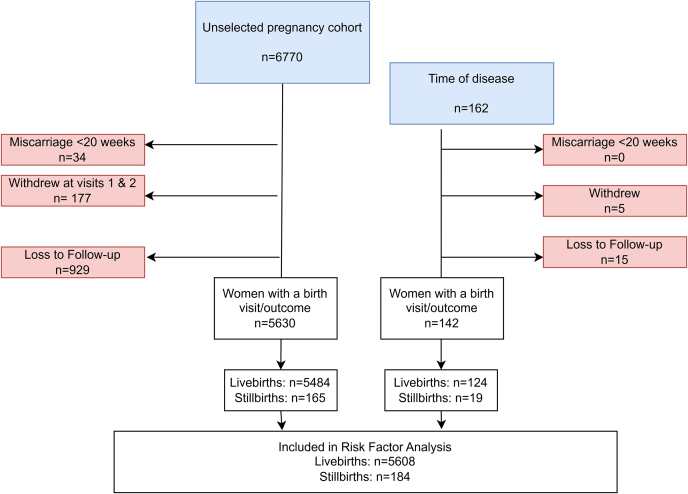
Study Flow diagram for the PRECISE stillbirth study, Kenya, Mozambique, and The Gambia, 2019–2022. Note: Unselected pregnancy cohort refers to women recruited to the PRECISE study at first ANC visit from recruiting clinics. The time of disease cohort includes women recruited to the PRECISE study after the onset of one or more placental disorders (pregnancy-induced hypertension, suspected fetal growth restriction or intra-uterine fetal death/stillbirth).

The study included 5772 women from both the unselected pregnancy and the time of disease cohorts, with 5608 livebirths and 184 stillbirths recorded (Fig. 2 and Supplementary Fig. S3).

The median maternal age at recruitment was 26.0 years, with 14.2% less than 20 and 11.8% over 35 years. Most mothers had at least primary education (81.0%), identified as housewives (67.0%), were married or cohabiting (82.0%), and had access to improved sanitation (79.4%) and at least limited water access (91.6%) (Table 1). Full participant characteristics including occupation, marital status and additional variables are presented in Supplementary Table S5-1. Maternal residence patterns varied by country: approximately two-thirds of participants in Kenya and Mozambique resided in urban areas, whereas a comparable proportion in The Gambia lived in rural settings. The median gestational age at recruitment and delivery was 20.9 (IQR: 16.3, 25.4) weeks and 39.3 (IQR: 29.3, 38.3) weeks for women with livebirths, and 19.0 (IQR: 14.1, 25.0) and 35.3 (IQR: 29.3, 38.3) for women with stillbirths. Most babies (82.0%) were delivered via unassisted vaginal cephalic delivery, with 92.5% of deliveries occurring in a health facility.

**Table 1:** Selected potential risk factors and their association with stillbirth (≥20 weeks) in the PRECISE cohort, pooled bivariate analysis–Unselected pregnancy cohort and time of disease cohort.

	Livebirths (N = 5608) n (%)	Stillbirths (N = 184) n (%)	Crude RR (95% CI)	p-value
Distal factors				
Maternal education				
None	1040 (18.5)	58 (31.5)	2.60 (1.24–5.45)	0.011
Primary/Secondary	4177 (74.5)	117 (63.6)	1.34 (0.66–2.75)	0.421
Higher	386 (6.9)	8 (4.3)	1 (Ref)	
Missing	5 (0.1)	1 (0.5)		
Village rural/urban index				
Urban/Peri-urban	3841 (68.5)	99 (53.8)	1 (Ref)	
Rural	1630 (29.1)	66 (35.9)	1.54 (1.13–2.11)	0.006
Missing	137 (2.4)	19 (10.3)		
Poverty likelihood, % (median, IQR)	21.4 (6.1, 42.3)	31.7 (8.5, 51.3)	1.01 (1.01–1.02)	<0.001
Intermediate factors				
Household sanitation access				
Improved	4467 (79.7)	132 (71.7)	1 (Ref)	
Unimproved	1115 (19.9)	50 (27.2)	1.50 (1.08–2.07)	0.015
Missing	26 (0.5)	2 (1.1)		
Household water access				
Improved/at least limited	5143 (91.7)	162 (88.0)	1 (Ref)	
Unimproved	309 (5.5)	16 (8.7)	1.61 (0.96–2.69)	0.068
Missing	156 (2.8)	6 (3.3)		
Cooking fuel source				
Biomass	2156 (38.4)	94 (51.1)	2.59 (1.50–4.47)	0.001
Coal	2512 (44.8)	74 (40.2)	1.78 (1.02–3.09)	0.043
Other (Battery/electric/gas/kerosene)	916 (16.3)	15 (8.2)	1 (Ref)	
Missing	24 (0.4)	1 (0.5)		
Conception season				
Dry	2259 (40.3)	86 (46.7)	1 (Ref)	
Wet	3167 (56.5)	75 (40.8)	0.63 (0.46–0.86)	0.004
Missing	182 (3.2)	23 (12.5)		
Maternal age (years)				
15–19	797 (14.2)	28 (15.2)	1.39 (0.86–2.25)	0.179
20–24	1639 (29.2)	41 (22.3)	1 (Ref)	
25–29	1510 (26.9)	48 (26.1)	1.26 (0.83–1.92)	0.273
30–34	1006 (17.9)	36 (19.6)	1.42 (0.90–2.22)	0.128
≥35	652 (11.6)	31 (16.8)	1.86 (1.17–2.97)	0.009
Missing	4 (0.1)	0 (0.0)		
Maternal BMI at recruitment				
<18.5	277 (4.9)	17 (9.2)	1.89 (1.13–3.16)	0.016
18.5–24.9	3099 (55.3)	98 (53.3)	1 (Ref)	
25–29.9	1494 (26.6)	47 (25.5)	0.99 (0.70–1.41)	0.977
≥30	715 (12.7)	20 (10.9)	0.89 (0.55–1.44)	0.627
Missing	23 (0.4)	2 (1.1)		
Parity				
1	1652 (29.5)	50 (27.2)	1.04 (0.73–1.47)	0.842
2–4	2981 (53.2)	87 (47.3)	1 (Ref)	
≥5	975 (17.4)	47 (25.5)	1.62 (1.14–2.31)	0.008
History of previous stillbirth				
No	5143 (91.7)	140 (76.1)	1 (Ref)	
Yes	409 (7.3)	42 (22.8)	3.51 (2.49–4.96)	<0.001
Missing	56 (1.0)	2 (1.1)		
Proximal factors				
American Heart Association category for blood pressure				
No hypertension	5001 (89.2)	142 (77.2)	1 (Ref)	
Stage 1 hypertension^a^	408 (7.3)	24 (13.0)	2.01 (1.31–3.10)	0.002
Stage 2 hypertension	192 (3.4)	18 (9.8)	3.10 (1.90–5.07)	<0.001
Missing	7 (0.1)	0 (0.0)		
Pre-eclampsia				
No	5162 (92.0)	153 (83.2)	1 (Ref)	
Yes	439 (7.8)	31 (16.8)	2.29 (1.56–3.37)	<0.001
Missing	7 (0.1)	0 (0.0)		
Antepartum haemorrhage				
No	5320 (94.9)	160 (87.0)	1 (Ref)	
Yes	288 (5.1)	24 (13.0)	2.64 (1.72–4.05)	<0.001
Placental abruption				
No	5593 (99.7)	179 (97.3)	1 (Ref)	
Yes	15 (0.3)	5 (2.7)	8.06 (3.32–19.60)	<0.001
Mode of delivery				
Unassisted vaginal/cephalic	4610 (82.2)	141 (76.6)	1 (Ref)	
Vaginal breech	137 (2.4)	9 (4.9)	2.08 (1.06–4.08)	0.033
Operative vaginal	147 (2.6)	5 (2.7)	1.11 (0.45–2.71)	0.821
Caesarean section	628 (11.2)	19 (10.3)	0.99 (0.61–1.60)	0.966
Missing	86 (1.5)	10 (5.4)		
Delivery location				
PHC/hospital	5207 (92.8)	152 (82.6)	1 (Ref)	
Outside health facility	326 (5.8)	25 (13.6)	2.51 (1.65–3.83)	<0.001
Missing	75 (1.3)	7 (3.8)		
Size for gestational age at birth				
Small for gestational age (<10th centile)	952 (17.0)	39 (21.2)	2.03 (1.38–3.00)	<0.001
Appropriate for gestational age (10th–90th Centile)	3644 (65.0)	72 (39.1)	1 (Ref)	
Large for gestational age (>90th centile)	338 (6.0)	4 (2.2)	0.60 (0.22–1.65)	0.326
Missing	674 (12.0)	69 (37.5)		
Birth plurality				
Singleton	5419 (96.6)	171 (92.9)	1 (Ref)	
Multiple^b^	189 (3.4)	13 (7.1)	2.10 (1.20–3.70)	0.010
Missing	0 (0.0)	0 (0.0)		
Sex of baby				
Male	2835 (50.6)	81 (44.0)	1.25 (0.90–1.74)	0.181
Female	2735 (48.8)	62 (33.7)	1 (Ref)	
Missing	38 (0.7)	41 (22.3)		

Numbers shown are pooled across the three PRECISE sites (Kenya, Mozambique, The Gambia). RR = unadjusted risk ratio from modified Poisson regression with robust standard errors. Reference categories are indicated by RR = 1 (Ref). Country-stratified counts and percentages for all variables, together with additional risk factors not retained for the multivariable model (occupation, marital status, distance to clinic, air exposure, delivery season, MUAC, HIV status, female genital mutilation, previous Caesarean section, tobacco use, gestational age at recruitment), are presented in Supplementary Table S5.1. BMI = body mass index; AHA = American Heart Association; PHC = primary health centre; IQR = interquartile range.

a Includes women with normal or elevated blood pressure.

b Includes twins only, there were no triplets or higher order births.

### Stillbirth rates

5630 women with 5484 livebirths and 165 stillbirths were from the PRECISE unselected pregnancy cohort giving an overall stillbirth rate at ≥20 weeks of 29.2 per 1000 total births (95%CI: 24.8–33.6) (Table 2). Stillbirth rates varied across sites and were significantly lower in Kenya (17.5 per 1000 total births (95% CI:12.5–22.5)), compared to Mozambique (32.8 (95% CI:24.7–40.9)), and The Gambia (49.8 (95%CI: 37.4–62.4)). Overall, the majority (89.1%) of stillbirths occurred at ≥28 weeks, ranging from 82.8% in The Gambia to 93.4% in Mozambique.

**Table 2:** Prevalence of stillbirth in the PRECISE unselected pregnant cohort.

	Kenya	Mozambique	The Gambia	Total
Total Births (≥20 weeks)	2625	1861	1163	5649
Total Stillbirths (≥20 weeks)	46	61	58	165
SBR ≥20 weeks (95% CI)	17.5 (12.5–22.5)	32.8 (24.7–40.9)	49.8 (37.4–62.4)	29.2 (24.8–33.6)
Total Births (≥22 weeks)	2625	1860	1162	5647
Total Stillbirths (≥22 weeks)	46	60	57	163
SBR ≥22 weeks (95% CI)	17.5 (12.5–22.5)	32.3 (24.2–40.3)	49.1 (36.6–61.4)	28.9 (24.5–33.2)
Total Births (≥28 weeks)	2621	1857	1153	5631
Total Stillbirths (≥28 weeks)	42	57	48	147
SBR ≥28 weeks (95% CI)	16.0 (11.2–20.8)	30.7 (22.9–38.5)	41.6 (30.1–53.1)	26.1 (21.9–30.3)
Percentage of all stillbirths (≥28 weeks)	91.3%	93.4%	82.8%	89.1%

SBR–Stillbirth rate, per 1000 total births.

Note: Denominators differ across gestational age thresholds as births occurring below each respective threshold are excluded from the relevant calculation.

### Factors associated with stillbirth

All variables had a VIF and correlation coefficients below thresholds and no variables were excluded based on collinearity.

Bivariable analysis identified several distal factors associated with increased stillbirth risk. Babies born to mothers without formal education had over twice the risk of stillbirth compared with those with higher/post-secondary education (RR 2.54, 95% CI 1.21–5.31, p = 0.013). Rural residence was linked to a 50% higher risk relative to urban/peri-urban residence (RR 1.50, 95% CI 1.11–2.04, p = 0.009). Additionally, each percentage point increase in the likelihood of living below the poverty line was associated with a rise in stillbirth risk (RR 1.01, 95% CI 1.01–1.02, p < 0.001).

Intermediate factors associated with increased stillbirth risk included lack of access to improved sanitation or water (RR 1.49, 95% CI 1.08–2.07, p = 0.016; RR 1.60, 95% CI 0.96–2.67, p = 0.073), use of coal or biomass as cooking fuel source during pregnancy (RR 1.76, 95%CI 1.01–3.06, p = 0.046; RR 2.56, 95%CI 1.49–4.41, p < 0.001, respectively), maternal age ≥35 years (RR 1.86, 95%CI 1.17–2.96, p = 0.009), body mass index (BMI) < 18.5 (RR 1.88, 95% CI 1.12–3.14, p = 0.017), high parity ≥5 (RR 1.62, 95%CI 1.14–2.31, p = 0.008), and previous stillbirth (RR 3.48, 95% CI 2.46–4.91, p < 0.001). Conceiving in the wet season was associated with a lower stillbirth risk (RR 0.68, 95%CI 0.50–0.92, p = 0.011).

Proximal factors associated with increased stillbirth risk included delivery outside of a health facility (RR 2.47, 95%CI 1.62–3.77, p < 0.001), antepartum haemorrhage (APH) (RR 2.63, 95%CI 1.72–4.05, p < 0.001), placental abruption (RR 8.06, 95%CI 3.31–19.6, p < 0.001), vaginal breech delivery (RR 2.03, 95% CI 1.04–3.99, p = 0.039), and being small-for-gestational-age (SGA) (RR 1.45, 95% CI 1.00–2.10, p = 0.050). There was a dose-dependent relationship between maternal hypertension and stillbirth risk (Stage 1 RR 2.01, 95% CI 1.31–3.10, p = 0.002; Stage 2 RR 3.10, 95% CI 1.90–5.07, p < 0.001), with increased risk also with pre-eclampsia (RR 2.29, 95%CI 1.56–3.37, p < 0.001).

In the adjusted analysis, significant risk factors were a history of previous stillbirth (aRR = 3.18, 95% CI: 1.85–5.46) Stage 2 hypertension (aRR = 3.25, 95% CI: 1.42–7.44), APH (aRR = 3.10, 95% CI: 1.77–5.42), Caesarean delivery (aRR = 1.74, 95%CI:1.01–3.01) and being born small-for-gestational age (aRR = 2.05 (95%CI:1.34–3.12)) (Table 3). The complete-case analytical sample for Model 3 comprised 4803 women (4702 livebirths and 101 stillbirths), distributed across Kenya (2180 women; 32 stillbirths), Mozambique (1752 women; 44 stillbirths), and The Gambia (871 women; 25 stillbirths). Sensitivity analyses building the multivariate model using the imputed dataset found similar results to those obtained from the complete case analysis, with strong evidence of increased risk associated with previous stillbirth, APH, and hypertension. Additionally, delivery outside a health facility was strongly associated with increased risk (aRR = 2.32, 95% CI: 1.49–3.62, p < 0.001) (Supplementary information S5-3). A further sensitivity analysis including country as a fixed effect in Model 3 yielded results consistent with the primary analysis for all five principal risk factors, with country itself not reaching statistical significance in the fully adjusted model (Supplementary information S5-5).

**Table 3:** Multivariable analysis of determinants of stillbirth (≥20 weeks) in the PRECISE cohort.

	Model 1: adjusted for distal factors Livebirth = 5469; stillbirths = 165		Model 2: adjusted for distal and intermediate factor Livebirth = 5356; stillbirths = 160		Model 3: adjusted for distal, intermediate and proximal factors Livebirth = 4702; stillbirths = 101	
	RR (95% CI)	p-value	RR (95% CI)	p-value	RR (95% CI)	p-value
Maternal education						
None	1.58 (0.71–3.52)	0.260	1.83 (0.77–4.31)	0.170	1.70 (0.54–5.31)	0.363
Primary	1.14 (0.54–2.41)	0.736	1.46 (0.65–3.25)	0.360	1.74 (0.61–5.01)	0.304
Secondary	0.94 (0.44–2)	0.866	1.12 (0.5–2.54)	0.780	1.23 (0.43–3.61)	0.697
Higher	1 (Ref)		1 (Ref)		1 (Ref)	
Village - rural and urban index						
Urban/Peri Urban	1 (Ref)		1 (Ref)		1 (Ref)	
Rural	1.29 (0.93–1.81)	0.129	1.22 (0.86–1.72)	0.257	1.03 (0.66–1.62)	0.865
%Likelihood below the poverty line, median (IQR)	1.01 (1 -1.02)	0.072	1.01 (1 -1.02)	0.145	1.01 (1 -1.02)	0.086
Previous stillbirth						
No			1 (Ref)		1 (Ref)	
Yes			3.35 (2.21–5.09)	<0.001^b^	3.18 (1.85–5.46)	<0.001^b^
Maternal BMI (categorised)						
<18.5			1.83 (1.07–3.12)	0.026	1.39 (0.62–3.15)	0.427
18.5–24.9			1 (Ref)		1 (Ref)	
25–29.9			1.13 (0.77–1.65)	0.528	1.40 (0.88–2.24)	0.155
≥30			0.9 (0.52–1.58)	0.726	1.14 (0.59–2.20)	0.689
Maternal age (categorised)						
15–19			1.41 (0.83–2.43)	0.207	1.60 (0.81–3.14)	0.173
20–24			1 (Ref)		1 (Ref)	
25–29			1.47 (0.91–2.38)	0.115	1.49 (0.81–2.74)	0.201
30–34			1.61 (0.92–2.81)	0.092	1.54 (0.76–3.11)	0.228
35+			2.06 (1.12–3.78)	0.019	1.92 (0.88–4.14)	0.097
Parity						
1			1.57 (0.99–2.49)	0.056	1.29 (0.73–2.29)	0.378
2–4			1 (Ref)		1 (Ref)	
5+			0.77 (0.47–1.26)	0.297	0.68 (0.36–1.28)	0.229
Conception season						
Dry			1 (Ref)		1 (Ref)	
Wet			0.69 (0.5–0.95)	0.024	0.78 (0.52–1.17)	0.234
HIV status						
No			1 (Ref)		1 (Ref)	
Yes			0.52 (0.21–1.27)	0.148	0.50 (0.19–1.32)	0.162
Sex of baby						
Male					1.06 (0.71–1.57)	0.784
Female					1 (Ref)	
American Heart Association category for blood pressure						
No hypertension^a^					1 (Ref)	
Stage 1 Hypertension					1.52 (0.80–2.87)	0.200
Stage 2 Hypertension					3.25 (1.42–7.44)	0.005^b^
Antepartum haemorrhage						
No					1 (Ref)	
Yes					3.10 (1.77–5.42)	<0.001^b^
Tobacco use						
No					1 (Ref)	
Yes					2.10 (0.51–8.69)	0.307
Preeclampsia						
No					1 (Ref)	
Yes					1.53 (0.83–2.82)	0.177
Mode of delivery						
Unassisted vaginal/Cephalic					1 (Ref)	
Operative vaginal					0.95 (0.29–3.10)	0.937
Vaginal breech					1.89 (0.79–4.54)	0.152
Caesarean section					1.74 (1.01–3.01)	0.046
Birth plurality						
Singleton					1 (Ref)	
Multiple					1.49 (0.73–3.07)	0.277
Size for gestational age						
Small for gestational age (<10th centile)					2.05 (1.34–3.12)	0.001^b^
Appropriate for gestational age (10th to 90th centile)					1 (Ref)	
Large for gestational age (>90th centile)					0.47 (0.15–1.52)	0.209
Akaike’s information criterion (AIC)	1485.14		1417.28		942.17	
Bayesian information criterion (BIC)	1524.96		1536.35		1136.48	

BMI= Body Mass Index, PHC = primary health centre, IQR – Interquartile range.

a Includes women with normal or elevated blood pressure. Data from both the unselected pregnancy cohort and the time of disease cohort. The complete-case analytical sample for Model 3 comprised 4702 livebirths and 101 stillbirths (Kenya: 2148 livebirths, 32 stillbirths; Mozambique: 1708 livebirths, 44 stillbirths; The Gambia: 846 livebirths, 25 stillbirths).

b p < 0.05.

## Discussion

Stillbirth remains an important public health challenge in these populations, with 1 in 34 women’s pregnancies ending in a stillbirth in this cohort. Our findings support multifactorial contributors to stillbirth, including maternal, obstetric, and healthcare-related risk factors. Maternal and fetal complications, including hypertension in pregnancy, APH, a previous history of stillbirth, and being born small-size-for gestational age at birth, a proxy for fetal growth restriction, were important drivers of stillbirth. Additionally, caesarean section was a risk factor for stillbirth, likely reflecting delays in timely identification of intrapartum complications and access to emergency obstetric care. These findings underscore the urgent need for integrated ANC strategies addressing the detection and management of clinical risk factors and the strengthening of referral pathways and facility-readiness for high-quality intrapartum monitoring and timely emergency obstetric care to address preventable stillbirths.

Despite recruitment at ANC clinics rather than in the community, high ANC utilisation (Kenya: 97.9%, Mozambique: 87.0%, The Gambia:97.8%) suggests the cohort is broadly representative of pregnant women in these areas.^22^ Large variations in stillbirth rates (≥20 weeks) were observed across the three populations, ranging from 17.5 per 1000 in Kenya to 49.8 in the Gambia. These differences may reflect epidemiological variation due to socio-demographic disparities: with The Gambian cohort being more rural (60.4%), with lower access to formal education (35.8%) and higher poverty levels compared to the other countries (Kenya is more urban (87.8%) and has higher access to formal education (91.3%) while in Mozambique 58.3% were urban residents and 94.5% had access to formal education).

However, as women experiencing stillbirth may have been disproportionately lost to follow-up, our estimates may underestimate the true stillbirth burden, especially in Kenya where follow-up was lowest. Additionally, between-site variations may also reflect variability in misclassification between stillbirths and early neonatal deaths, especially at lower gestational ages. Disparities in resuscitation practices can exacerbate misclassification, for example Helping Babies Breathe training is associated with a 38% reduction in intrapartum stillbirth through improved newborn assessment and resuscitation of non-macerated newborns without vital signs at birth.^23^ Coverage of neonatal resuscitation was lowest in The Gambia facilities where stillbirth rates were highest suggesting possible misclassification in some cases.

In high-income countries, around a third of all stillbirths occur between 20^+0^ to 27^+6^, in contrast to 10.9% in this study, ranging from 8.7% in Kenya to 17.2% in The Gambia.^24^ Despite efforts to ensure early recruitment in pregnancy, only 50% of participants were enrolled before 20 weeks (Kenya: 43.5%; The Gambia: 45.3%; Mozambique: 51.9%), potentially missing early stillbirths if intrauterine fetal death occurred prior to enrolment. Due to challenges during the COVID-19 pandemic and Kenyan nurses’ strikes, pregnancy outcomes were available for 82.9% of the cohort (The Gambia: 95.4%; Mozambique: 89.0%; Kenya: 74.8%).

Due to challenges in accurately capturing early gestation stillbirths, UN global estimates currently include only late gestation stillbirths (≥28 weeks).^3^ Late gestation stillbirth rates in this study were lower than 2021 UN estimates for Kenya (16.0 (95%CI:11.2–20.8) vs. 18.1 (UI:17.2–19.1)), but substantially higher for Mozambique (30.7 (95%CI:22.9–38.5) vs. 18.3 (UI:14.9–22.3)) and The Gambia (41.6 (95%CI:30.1–53.1) vs. 18.9 (UI:12.1–29.9)).^3^ These discrepancies may reflect socio-demographic differences between our study cohort and national populations, for example in The Gambia much lower stillbirth rates have been reported in coastal urban areas.^25^ Additionally, potential underestimation in UN global estimates due to data sparsity (e.g., only one study informed estimates for The Gambia and Mozambique, vs. multiple sources for Kenya) could further contribute to these differences.

Similar to previous African studies, we found a previous history of stillbirth, hypertension in pregnancy, APH, being small-for-gestational age and birth by caesarean section to be important risk factors for stillbirth in the adjusted analyses.^3^^,^^9^^,^^10^^,^^26^

Prior stillbirth was associated with a more than threefold increased risk. This association may be explained by the recurrence of underlying maternal conditions that persist across pregnancies, including chronic hypertension, diabetes mellitus, haemoglobinopathy (sickle cell) and thrombophilia. Women with a prior stillbirth may also face shared socioeconomic or healthcare access barriers that increase their risk of subsequent adverse outcomes. Post-stillbirth care should incorporate efforts to detect and manage underlying maternal conditions and counselling for future pregnancy planning, alongside pre-conception strategies to optimise health (e.g., nutritional support, chronic disease control, and early antenatal engagement with individualised risk monitoring and psychological support).^27^

Hypertensive disorders of pregnancy remain an important driver of adverse outcomes with severe hypertension associated with three times the risk of stillbirth in this study. Both gestational hypertension and pre-eclampsia are associated with uteroplacental mismatch, and share poor placentation as an underlying pathology with stillbirth when occurring remote from term.^28^ These data complement the findings from the Community-Level Interventions for Pre-eclampsia (CLIP) trials that were completed in India, Mozambique, and Pakistan.^29^ In CLIP intervention arms, Stage 1 hypertension was associated with a trend towards more stillbirth (RR 1.24 (0.97–1.58)), while non-severe and severe Stage 2 hypertension were associated with increased risks of stillbirth (RR 1.81 (1.30–2.54); 5.88 (3.95–8.73), respectively).^30^ It is unclear whether or not calcium can reduce the burden of pre-eclampsia in women with inadequate intakes.^31^^,^^32^ The detection of pre-eclampsia is a signal function of ANC. Complete assessment of women who have been diagnosed with pregnancy hypertension can identify those women at increased risk of stillbirth, providing the opportunity to intervene prior to the fetal loss.^33^

APH compromises placental oxygen exchange and may lead to acute fetal decompensation, and where there are delays in recognising APH or accessing emergency obstetric care may result in fetal demise. It is an important potentially modifiable risk factor for stillbirth, associated in this study, with a more than three-fold increase in risk. Improved antenatal surveillance of early detection and management of risk factors including hypertension, smoking, and trauma (e.g., gender-based violence and placental praevia), coupled with increasing awareness amongst pregnant women and providers and strengthened emergency referral services could mitigate haemorrhage-related complications and improve perinatal outcomes.^34^

In contrast to many previous African studies, we were able to assess the association between size-for-GA and stillbirth. The association between small-for-gestational age (SGA) and stillbirth is biologically plausible and likely mediated by chronic placental insufficiency, which renders the fetus more susceptible to hypoxic injury during labour. SGA may also reflect underlying maternal nutritional deficiencies, infection, or vascular pathology that independently increase stillbirth risk. However, the SGA-stillbirth association differed by analytic approach: complete-case analysis yielded a two-fold increased risk (aRR = 2.05; 95% CI: 1.34–3.12), while multiple imputation produced an attenuated, non-significant estimate (aRR = 1.33; 95% CI: 0.92–1.92). This divergence reflects competing biases. Complete-case excludes stillbirths with missing data, while imputation violates the missing-at-random assumption (birthweight was differentially missing for stillbirths (37.5% vs. 12.0% for livebirths)) biasing imputed values toward the normal range and diluting the association. Adding further complexity, while SGA remains a critical, readily available indicator, the static nature of SGA measured at birth inadequately reflects FGR’s dynamic pathophysiology, particularly in stillbirths: maceration reduces fetal weight, while intrauterine retention creates discordance between size at death and birth, inflating SGA rates and potentially overestimating the true FGR-stillbirth association.^35^ Dating heterogeneity compounds this: only 31.1% of Gambian women had ultrasound-based gestational age assignment compared with 87.6% in Mozambique (Supplementary Table S2.2), so country differences in the size-for-gestational-age distribution should be interpreted with caution. Given biological plausibility, we consider the complete-case estimate the more defensible reflection of the true association, though both estimates may be subject to bias in opposite directions. This underscores the urgent need for research and implementation of better prenatal biomarkers (e.g., serial fundal height, ultrasound Doppler) to identify and manage true FGR before fetal demise occurs.

Birth by caesarean section emerged as a risk factor, which warrants careful interpretation. In the settings of this study, caesarean delivery is frequently performed in response to obstetric emergencies such as non-reassuring fetal status, obstructed labour, or severe maternal complications. Thus, caesarean section in this context is more appropriately viewed as a marker of underlying intrapartum compromise rather than a direct causal factor for stillbirth. This distinction is critical to avoid misinterpretation of the directionality of this association.

Our findings differed from prior studies which have reported associations with maternal (socio-economic status, education, age, parity, anaemia), fetal (malpresentation, gestational age at birth, birthweight, twins, male sex, congenital anomalies) and healthcare factors (suboptimal antenatal/intrapartum care, referral delays, birth outside of a health facility) and stillbirth.^3^^,^^6^^,^^9^^,^^10^ In this study poverty, lack of education, advanced maternal age and grand multiparity whilst strongly associated with stillbirth risk in the bivariable analysis their effect was attenuated in adjusted models suggesting mediation through proximal factors. We were unable to assess anaemia in this study.

No information was available regarding fetal presentation: however, vaginal breech delivery occurred in 2.5% of births and was associated with a higher stillbirth risk than cephalic vaginal delivery on bivariable analysis (RR 2.03, 95% CI 1.04–3.99). While the significance of association was attenuated in the fully adjusted model (RR 1.89, 95% CI 0.79–4.54), prior studies have found breech presentation to be a risk factor for stillbirth with careful obstetric management recommended.^36^^,^^37^ In contrast to meta-analysis findings of a 10% elevated stillbirth risk in males, we found no strong evidence of a difference in risk by sex.^38^ Despite a twofold increased risk in bivariate analysis, twins were not retained in the adjusted model suggesting the increased risk could be mediated through twin-associated pregnancy complications including hypertension, fetal growth restriction and APH associated with placentation disorders.^39^ These findings underscore the importance of early identification of multiple pregnancies and enhanced antenatal monitoring for these complications, particularly in monochorionic twins.^40^

In terms of healthcare factors, most stillbirths occurred preterm, precluding completion of routine ANC, which could conflate the association between number of ANC visits and stillbirth. We therefore did not look at the association with number of ANC. Gestational age at enrolment was slightly lower for women with stillbirth median 19.0 weeks, compared to 20.9 weeks for livebirths, although this may reflect earlier ANC attendance for women with previous poor obstetric history, or complications in the current pregnancy, both of which increase stillbirth risk.

Overall, this study’s findings can inform actionable insights for health service delivery. The high baseline stillbirth risk and persistent importance of conditions like hypertension and fetal growth restriction in this population of women who have successfully accessed ANC underscores need for the scale-up of effective evidence-based ANC packages including reliable access to blood pressure monitoring, ultrasound for fetal growth assessment, and aspirin for pre-eclampsia prevention in high-risk women. The association with caesarean section and APH underscores a critical need to strengthen the continuum of care requiring policies that invest in emergency obstetric care and standardised referral protocols. Finally, the elevated risk following a previous stillbirth highlights the necessity for targeted follow-up and supportive care for bereaved mothers in subsequent pregnancies.

This study’s strengths include its multi-country prospective design (5600 births across three geographically diverse African populations recruited at first ANC attendance), application of a conceptual framework to model structural, intermediate, and proximal risk factors for stillbirth sequentially, and novel identification of SGA births as a key risk factor, a critical gap in African perinatal research. While our ANC-based design excludes the highest-risk women whose pregnancies end before the first visit or who never access care, the exceptionally high ANC attendance in our study settings means our cohort captures a more representative population base than many previous facility-based studies in Africa, which have often relied on single-site recruitment exclusively at the time of stillbirth. Whilst we were unable to undertake first trimester ultrasound dating scans on all participants, we employed a systematic approach towards the best obstetric estimate of gestational age for each woman with 59.1% overall having an ultrasound or Tracer based GA assessment.

This study had important limitations. First, despite the large overall cohort, site-specific variability in stillbirth rates and the limited number of stillbirths (n = 184) constrained subnational analyses and reduced power to detect rarer associations. Second, for the risk factor analysis, we combined the prospective cohort with a ‘time-of-disease’ cohort to augment the number of stillbirth cases. While this improved model precision, the resulting case-enriched sampling frame means the reported risk ratios should be interpreted as robust indicators of important determinants within similar healthcare settings rather than as unbiased, population-level effect estimates. Third, in line with the primary aim of identifying individual-level risk factors, our models did not adjust for clustering at the country or health-facility level. During model development, country was found to be strongly correlated with key distal exposures (rurality, r = 0.47; poverty, r = 0.41), and its inclusion absorbed the effects of these variables. A sensitivity analysis including country as a fixed effect in the fully adjusted model confirmed that the principal associations were robust, with all five key risk factors retaining consistent magnitude and significance, while country itself was not statistically significant. The country distribution of the complete-case analytical sample is reported in the Results. We acknowledge that our estimates do not account for contextual variance, such as unmeasured differences in facility-based quality of care, which may confound associations, particularly for clinical management factors (e.g., caesarean section). Although ethnicity was collected, the site-specific and granular categories did not support analysis of within-country ethnic variation in stillbirth risk. Finally, while our incremental modelling approach minimized overadjustment, it may not fully resolve residual confounding inherent to observational designs.

While our analytical framework structured risk factors hierarchically, the relationships are likely interactive and reciprocal. Our findings should thus be interpreted as revealing points of intervention within a complex, interdependent system.

Additionally, although we evaluated multiple potential risk factors, others such as sleeping position, chronic medical conditions, infectious diseases, interpregnancy period were not examined.^9^^,^^41^ Additionally, anaemia status, initially planned for assessment, could not be evaluated due to inaccuracies in the Masimo device used.^42^ Intrapartum stillbirth rate an important marker of quality of intrapartum care, will be examined in future analyses.^3^

Almost two-thirds of stillbirths (63.0%) in this study were born preterm. Further research is needed to understand drivers of preterm stillbirths and inform context-specific interventions, including quantifying further the role of other associated co-morbidities associated with both antepartum fetal death and preterm labour such as malaria, and gestational diabetes.^43^

Building upon the approach of the Fetal Medicine Foundation, there may be an opportunity to combine maternal factors (e.g., maternal age, parity, previous stillbirth, medical conditions), clinical signs (e.g., blood pressure), biomarkers (e.g., PlGF), and health system factors (e.g., per capita gross domestic product), into either a competing risks-based or machine learning-based tool to identify individual women at increased risk of stillbirth at antenatal booking and follow-up visits so that they can be offered increased monitoring and timed birth to defray those risks.^44^^,^^45^

### Conclusion

Stillbirth remains a critical public health challenge in these populations, with large country-level variation in rates. This study’s findings highlight a multifactorial stillbirth aetiology driven by maternal and fetal complications (hypertensive disorders and small-size-for gestational age at birth, a proxy for fetal growth restriction), obstetric complications (APH), and healthcare challenges (increased risk with delivery by caesarean section potentially reflecting delays in timely identification of intrapartum complications and access to emergency obstetric care). Additionally, previous stillbirth remains an important marker of risk. Health systems must prioritise equitable access to quality evidence-based antenatal and intrapartum care, including standardised management of co-morbidities, timely risk identification, and dedicated post-stillbirth services with care for next-pregnancy to accelerate progress towards ending preventable stillbirths.

## Contributors

GM and AK contributed to conceptualization, data curation, formal analysis, investigation, methodology, project administration, supervision, and validation. JA contributed to data curation, formal analysis, investigation, methodology, software, and visualization. HJ, GO, MV and RC contributed to conceptualization, data curation, investigation, methodology, project administration and supervision. SM, AV contributed to Investigation, methodology, project administration, and supervision. MM contributed to data curation and investigation. SS and BA contributed to investigation and methodology. AR, UdA, ES, LM, RC, MT, PvD contributed to conceptualization, funding acquisition, investigation, methodology, and supervision. HB contributed to conceptualization, formal analysis, funding acquisition, investigation, methodology, supervision, and validation. GM, AK, JA and HB wrote the original draft. All authors contributed to the revision and editing of the manuscript and approved the final version. JA and HB have accessed and verified the data. HB and PvD were responsible for the decision to submit the manuscript.

## Data sharing statement

Data are available on reasonable request to the corresponding author. A fully searchable data dictionary and variable search tool providing details of all the data that are available can be found at https://precisenetwork.org/

## Declaration of interests

The authors have declared that no competing interests exist.

## Appendix A Supplementary data

Supplementary informations
