# Supplementary material for: Incidence and risk factors for stillbirth in Kenya, Mozambique and the Gambia: the PRECISE cohort study
Source: Lancet Reg Health Afr. 2026 Aug;6:None. doi: 10.1016/j.lanafr.2026.100089 (PMC13473389; doi:10.1016/j.lanafr.2026.100089)
Supplement: Supplementary file 1 [file mmc1.docx]

**PAPER TITLE**

Incidence and risk factors for stillbirth in Kenya, Mozambique and The Gambia: the PRECISE cohort study

**PAPER RUNNING TITLE**

Stillbirth: prevalence and risk factors in PRECISE

**SUPPORTING INFORMATION**

Contents

[S1: STROBE checklist	2](#_Toc230258886)

[S2 Assessment of exposure and outcome variables	5](#_Toc230258887)

[S2·1 Gestational age assessment	5](#_Toc230258888)

[S2·2 Method of Gestational Age assignment by country	7](#_Toc230258889)

[S2·3 Descriptions and assessment methods of potential determinants	8](#_Toc230258890)

[S3: Study participant flow diagrams by country	11](#_Toc230258891)

[S4 Multivariate model – assessment of collinearity	14](#_Toc230258892)

[S5 Bivariate and multivariate analyses using complete cases and imputed data	17](#_Toc230258893)

[Table S5·1 Potential risk factors and their association with stillbirth (≥20 weeks) in the PRECISE cohort, bivariate analysis by country and overall. – Unselected pregnancy cohort and time of disease cohort	17](#_Toc230258894)

[Table S5·2 Bivariate analysis of determinants of stillbirth	21](#_Toc230258895)

[Table S5·3 Bivariate analysis of additional factors associated with stillbirth (complete case analyses)	24](#_Toc230258896)

[Table S5·4 Multivariable analysis of risk factors for stillbirths ≥20 weeks (Imputed dataset analysis)	26](#_Toc230258897)

[Table S5·5 Sensitivity analysis: Multivariable model (Model 3) with and without country as a covariate (complete-case analysis)	28](#_Toc230258898)

[S6 Association between stillbirth and gestational age at birth (complete case analysis)	31](#_Toc230258899)

# S1: STROBE checklist

STROBE Statement—Checklist of items that should be included in reports of ***cohort studies***

|  | **Item No** | **Recommendation** |  |
| --- | --- | --- | --- |
| **Title and abstract** | 1 | (*a*) Indicate the study’s design with a commonly used term in the title or the abstract | Line 2 |
|  |  | (*b*) Provide in the abstract an informative and balanced summary of what was done and what was found | Lines 38 - 56 |
| **Introduction** | | |  |
| Background/rationale | 2 | Explain the scientific background and rationale for the investigation being reported | Lines 114-144 |
| Objectives | 3 | State specific objectives, including any prespecified hypotheses | Lines 146-148 |
| **Methods** | | |  |
| Study design | 4 | Present key elements of study design early in the paper | Lines 152-156 |
| Setting | 5 | Describe the setting, locations, and relevant dates, including periods of recruitment, exposure, follow-up, and data collection | Lines 151-189 |
| Participants | 6 | (*a*) Give the eligibility criteria, and the sources and methods of selection of participants. Describe methods of follow-up | Lines 160-174 |
|  |  | (*b*) For matched studies, give matching criteria and number of exposed and unexposed | NA |
| Variables | 7 | Clearly define all outcomes, exposures, predictors, potential confounders, and effect modifiers. Give diagnostic criteria, if applicable | Lines 191-213 |
| Data sources/ measurement | 8* | For each variable of interest, give sources of data and details of methods of assessment (measurement). Describe comparability of assessment methods if there is more than one group | Lines 191-213 |
| Bias | 9 | Describe any efforts to address potential sources of bias | Lines 178-187 |
| Study size | 10 | Explain how the study size was arrived at | Lines 171-174 |
| Quantitative variables | 11 | Explain how quantitative variables were handled in the analyses. If applicable, describe which groupings were chosen and why | Lines 191 - 213 |
| Statistical methods | 12 | (*a*) Describe all statistical methods, including those used to control for confounding | Lines 215-245 |
|  |  | (*b*) Describe any methods used to examine subgroups and interactions | Lines 226-228 |
|  |  | (*c*) Explain how missing data were addressed | Lines 228-232 |
|  |  | (*d*) If applicable, explain how loss to follow-up was addressed | - |
|  |  | (*e*) Describe any sensitivity analyses | Lines 231-233 |
| **Results** | | |  |
| Participants | 13* | (a) Report numbers of individuals at each stage of study—eg numbers potentially eligible, examined for eligibility, confirmed eligible, included in the study, completing follow-up, and analysed | Lines 255-256 Figure 2, Figure S3 |
|  |  | (b) Give reasons for non-participation at each stage | Figure 2 |
|  |  | (c) Consider use of a flow diagram | Figure 2 |
| Descriptive data | 14* | (a) Give characteristics of study participants (eg demographic, clinical, social) and information on exposures and potential confounders | Lines 255-266 |
|  |  | (b) Indicate number of participants with missing data for each variable of interest | Table 2 |
|  |  | (c) Summarise follow-up time (eg, average and total amount) | Lines 176-177 |
| Outcome data | 15* | Report numbers of outcome events or summary measures over time | Lines 268-274 |
| Main results | 16 | (*a*) Give unadjusted estimates and, if applicable, confounder-adjusted estimates and their precision (eg, 95% confidence interval). Make clear which confounders were adjusted for and why they were included | Lines 268 - 312 |
|  |  | (*b*) Report category boundaries when continuous variables were categorized | Table 2,3 |
|  |  | (*c*) If relevant, consider translating estimates of relative risk into absolute risk for a meaningful time period | - |
| Other analyses | 17 | Report other analyses done—eg analyses of subgroups and interactions, and sensitivity analyses | Lines 305-312 |
| **Discussion** | | |  |
| Key results | 18 | Summarise key results with reference to study objectives | Lines 316-325 |
| Limitations | 19 | Discuss limitations of the study, taking into account sources of potential bias or imprecision. Discuss both direction and magnitude of any potential bias | Lines 479-505 |
| Interpretation | 20 | Give a cautious overall interpretation of results considering objectives, limitations, multiplicity of analyses, results from similar studies, and other relevant evidence | Lines 522-531 |
| Generalisability | 21 | Discuss the generalisability (external validity) of the study results | Lines 471-477 |
| **Other information** | | |  |
| Funding | 22 | Give the source of funding and the role of the funders for the present study and, if applicable, for the original study on which the present article is based | Lines 566-572 |

# S2 Assessment of exposure and outcome variables

## S2·1 Gestational age assessment

For each woman, GA was assessed using a hierarchy based on the reliability of the method used, with routine ultrasound-based dating being prioritised if available. When foetal ultrasound measurements were available, GA was calculated from the most accurate method, namely foetal crown rump length (CRL) before 14 weeks^[1]^, or HC and femur length using the INTERGROWTH standards between 14 and 24 weeks^[2]^; if not available, GA was calculated from the estimated due date based on available routine ultrasound. When ultrasound was unavailable, the TraCer app was used to estimate GA based on foetal HC and TCD, and if Tracer was unavailable, GA estimates were based on last menstrual period (LMP). Finally, estimates based on SFH were only used for women with no other method available as this appears inferior to the methods above.^[3]^ GA at delivery of ≥43^+0^ weeks was considered implausible. For any given method, where GA at delivery was ≥43^+0^ weeks, the estimate was excluded and the estimate for the next method from the hierarchy was preferentially used.

Since LMP relies on recall, which may be inaccurate, we reviewed all participants dated by LMP to identify potential errors. Where the GA by LMP and SFH differed by more than 14 days, we introduced birthweight as a guide to accuracy; LMP was considered plausible where the birthweight fell with 2 standard deviations of the 50^th^ centile of birthweight for GA and sex, this was used for final GA assessment. Otherwise, the SFH-based estimate was assessed to determine if this yielded a more plausible GA estimate

Figure S2·1 Hierarchy Flow Chart for gestational age assessment

## S2·2 Method of Gestational Age assignment by country

|  | **Kenya** | **Mozambique** | **The Gambia** | **Total** |
| --- | --- | --- | --- | --- |
| US (EDD by biometry measurements)* | 248 (9·0%) | 1301 (69·2%) | 9 (0·7%) | 1558 (26·6%) |
| US (EDD by US machine) | 68 (2·5%) | 346 (18·4%) | 367 (30·4%) | 781 (13·4%) |
| TraCer | 818 (29·7%) | 0 (0·0%) | 309 (25·6%) | 1127 (19·3%) |
| LMP | 1174 (42·6%) | 190 (10·1%) | 280 (23·2%) | 1644 (28·1%) |
| SFH | 391 (14·2%) | 36 (1·9%) | 199 (16·5%) | 626 (10·7%) |
| No GA assigned | 38 (1·4%) | 3 (0·2%) | 30 (2·5%) | 71 (1·2%) |
| Outliers | 22 (0·8%) | 4 (0·2%) | 15 (1·2%) | 41 (0·7%) |

*Note: Outliers defined as births where gestational age at delivery was ≥43+0 weeks across all available estimation methods (see Figure S2.1 and accompanying text for the gestational age assessment hierarchy)*

## S2·3 Descriptions and assessment methods of potential determinants

| **Potential determinant** | **Method of assessment** | **Details** |
| --- | --- | --- |
| Maternal education | Questionnaire at recruitment |  |
| Religion | Questionnaire at recruitment |  |
| Marital Status | Questionnaire at recruitment |  |
| Occupation | Questionnaire at recruitment |  |
| Poverty Index | Calculated | Country-specific set of Progress out of poverty index (PPI) indicators and assigned scorecards and look up tables were obtained used to generate likelihoods and rates. <https://www.povertyindex.org/ppi-country>  PPI indicators, scorecards and look-up tables for 2005 and 2008 were used for Kenya and Mozambique respectively. For Gambia, a proxy from Senegal 2005 was used because Gambia does not have published PPIs. |
| Residence: Urban/ rural | Calculated | The degree of urbanization at address of residence calculated based on the Global Human Settlement Layer <https://human-settlement.emergency.copernicus.eu/> |
| Distance from clinic | Calculated | Calculated based on distance between reported address at recruitment and recruiting clinic |
| Water, Sanitation and Hygiene (WASH) variables | Calculated | Water sources and sanitation facilities were categorised into improved and unimproved using the WHO/UNICEF Joint Monitoring Programme (JMP) definitions^[4]^ based on questions from questionnaire at recruitment |
| Cooking fuel | Questionnaire at recruitment |  |
| Seasonality | Calculated | Binary variable 'wet' or 'dry' as derived from long term monthly climatic weather patterns. Dates of seasonal changes are the same year to year and  always fall on the first day of the month. Gambia and Mozambique have one 'wet' season per year. Kenya has two 'wet' seasons per year. |
| Air Pollution |  | Personal PM₂.₅ exposure was derived from high-resolution monitoring of 337 women (537 person-visits) across the 3 countries, with values categorised using UK and WHO guidelines. A random forest model, trained on demographic, behavioral, and environmental predictors, generalised these exposures to the larger PRECISE cohort, enabling population-level risk assessment. |
| Maternal Age | Questionnaire at recruitment |  |
| Maternal Body Mass Index (BMI) | Calculated from direct measurements at recruitment | Calculated based on average height and weight measures:  weight (kg)/ height (m)^2^ |
| Maternal Mid Upper Arm Circumference | Direct measurement at recruitment | Average mid upper arm circumference measured at recruitment visit |
| HIV | Questionnaire at recruitment/ Laboratory results | Assessed as positive if reported as currently on or initiated on antiretroviral medicine or with a positive HIV lab result |
| Female genital mutilation | Clinical records at labour visit |  |
| Parity | Calculated at delivery based on questionnaire at recruitment |  |
| Previous stillbirth | Questionnaire at recruitment |  |
| Previous C-section | Questionnaire at recruitment |  |
| Tobacco Use | Questionnaire at recruitment | Includes smoked, chewed or vaginal |
| Gestational Age at first ANC (antenatal clinic) visit | Calculated | Calculated based on final best GA assessment and date of first ANC (recruitment) visit |
| Hypertension | Direct measurement | Average of two readings is taken. If the first two reading differ >10mm/Hg, 3rd BP measurement is taken. Average of reading 2 and 3 is taken.  Coded according to American heart Association classification^[5]^:  Normal: systolic <120 AND diastolic <80  Elevated BP: systolic 120 – 129 AND diastolic <80)  Hypertension stage 1: systolic 130 – 139 OR diastolic 80 – 89  Hypertension stage 2: systolic ≥140 OR diastolic ≥90. |
| Pre-eclampsia | Calculated | Pre-eclampsia was diagnosed according to International Society for the Study of Hypertension in Pregnancy (ISSHP) criteria as chronic or gestational hypertension accompanied by either new-onset proteinuria (≥2+ on urinary dipstick or ≥30mg/mmol protein-to-creatinine ratio) or a pre-eclampsia-related complication. Complications included maternal end-organ dysfunction (e.g., neurological, hepatic, or hematological abnormalities) or uteroplacental dysfunction (e.g., fetal growth restriction). |
| Antepartum haemorrhage | Clinical records at labour visit |  |
| Placental Abruption | Clinical records at labour visit |  |
| Fetal growth restriction | Calculated | Estimated using the proxy of size <10^th^ Centile for Gestational Age according to Intergrowth-21^st^ charts^[6]^ |
| Fetal Sex | Assessment at birth |  |
| Gestational Age at Birth | Calculated | See S2 for full details |

# S3: Study participant flow diagrams by country

Figure S3·1 Kenya Flow Figure

Figure S3·2 Mozambique Flow Figure

Figure S3·3 The Gambia Flow Figure

# S4 Multivariate model – assessment of collinearity

We assessed multicollinearity using variance inflation factors (VIF) and pairwise correlation coefficients (Supplementary Information S4). All VIF values were below 10 (mean VIF: 2.02). Pairwise correlations exceeding 0.5 occurred only between continuous and categorical forms of the same variable; only one form was entered into the multivariable models, and no variables were excluded based on collinearity.

a. Variance Inflation Factor (VIF)

| **Variable** | **VIF** | **1/VIF** |
| --- | --- | --- |
| Highest school level |  |  |
| 1 | 3·81 | 0·262265 |
| 2 | 5·01 | 0·199581 |
| 3 | 4·93 | 0·202929 |
| Urban | 1·24 | 0·807743 |
| Poverty line | 1·61 | 0·621022 |
| 1.previous stillbirth | 1·11 | 0·900457 |
| Maternal BMI categories |  |  |
| 2 | 7·47 | 0·133894 |
| 3 | 7·67 | 0·130436 |
| 4 | 5·7 | 0·175569 |
| Maternal age |  |  |
| 2 | 2·47 | 0·404644 |
| 3 | 2·98 | 0·3355 |
| 4 | 2·87 | 0·348743 |
| 5 | 2·56 | 0·391272 |
| Parity |  |  |
| 1 | 2·09 | 0·478391 |
| 2 | 2·75 | 0·364093 |
| Season at conception | 1·09 | 0·915647 |
| HIV status | 1·08 | 0·924525 |
| Gestational age (categorised) |  |  |
| 1 | 1·09 | 0·916602 |
| 2 | 1·04 | 0·9653 |
| 3 | 1·12 | 0·89396 |
| 5 | 1·04 | 0·959262 |
| Sex | 1·01 | 0·986345 |
| Pacenta abruption | 1·07 | 0·93771 |
| Hypertension |  |  |
| 4 | 1·07 | 0·938095 |
| 5 | 1·15 | 0·86746 |
| Antepartum haemorrhage | 1·11 | 0·903587 |
| Tobacco use | 1·05 | 0·950795 |
| Mode |  |  |
| 2 | 1·07 | 0·937899 |
| 3 | 1·1 | 0·908554 |
| 4 | 1·12 | 0·890307 |
| Small for Gestational Age |  |  |
| 0 | 1·09 | 0·921578 |
| 3 | 1·09 | 0·917106 |
| pm25_3 |  |  |
| 2 | 1·78 | 0·560333 |
| 3 | 1·69 | 0·593174 |
| Average MUAC (categorised) |  |  |
| 2 | 4 | 0·249766 |
| 3 | 4·55 | 0·219851 |
| 4 | 5·29 | 0·189128 |
| Water | 1·08 | 0·922972 |
| Sanitation | 1·18 | 0·846995 |
| Religion |  |  |
| 2 | 1·7 | 0·589549 |
| 90 | 1·05 | 0·950941 |
| Marital status |  |  |
| 2 | 1·43 | 0·698957 |
| 3 | 1·08 | 0·92422 |
| Occupation |  |  |
| 2 | 1·32 | 0·755571 |
| 3 | 1·27 | 0·789864 |
| 9 | 1·11 | 0·899655 |
| 90 | 1·04 | 0·965299 |
| Sleeping position |  |  |
| 2 | 1·4 | 0·713653 |
| 3 | 1·39 | 0·717012 |
| Season at delivery | 1·08 | 0·922746 |
| Delivery location | 1·02 | 0·97595 |
| 1.pe_overall | 1·16 | 0·860065 |
| Female genital mutilation | 1·54 | 0·647331 |
| Distance to health facility (at booking visit) | 1·02 | 0·977308 |
| pm2.5 | 1·28 | 0·77929 |
| Twins | 1·07 | 0·932374 |
|  |  |  |
| Mean VIF | 2·02 |  |

b. Correlation Matrix – (Separate supplementary document available)

# S5 Bivariate and multivariate analyses using complete cases and imputed data

## Table S5·1 Potential risk factors and their association with stillbirth (≥20 weeks) in the PRECISE cohort, bivariate analysis by country and overall. – Unselected pregnancy cohort and time of disease cohort

|  | **KENYA** | | **MOZAMBIQUE** | | **THE GAMBIA** | | **TOTAL** | | **Association with stillbirth** | |
| --- | --- | --- | --- | --- | --- | --- | --- | --- | --- | --- |
|  | **livebirth** | **stillbirth** | **livebirth** | **stillbirth** | **livebirth** | **stillbirth** | **livebirth** | **stillbirth** | **RR (95% CI)** | **P-value** |
| **DISTAL FACTORS** | **N=2682** | **N=62** | **N=1800** | **N=61** | **N=1126** | **N=61** | **N=5608** | **N=184** |  |  |
| **Maternal Education** |  |  |  |  |  |  |  |  |  |  |
| None | 224 (8·4%) | 9 (14·5%) | 98 (5·4%) | 5 (8·2%) | 718 (63·8%) | 44 (72·1%) | 1040 (18·5%) | 58 (31·5%) | 2·60(1·24 -5·45) | 0·011** |
| Primary/Secondary | 2134 (79·6%) | 47 (75·8%) | 1683 (93·5%) | 55 (90·2%) | 360 (32·0%) | 15 (24·6%) | 4177 (74·5%) | 117 (63·6%) | 1·34(0·66 -2·75) | 0·421 |
| Higher | 319 (11·9%) | 5 (8·1%) | 19 (1·1%) | 1 (1·6%) | 48 (4·3%) | 2 (3·3%) | 386 (6·9%) | 8 (4·3%) | 1(Ref) |  |
| Missing | 5 (0·2%) | 1 (1·6%) | 0 (0·0%) | 0 (0·0%) | 0 (0·0%) | 0 (0·0%) | 5 (0·1%) | 1 (0·5%) |  |  |
| **Marital status** |  |  |  |  |  |  |  |  |  |  |
| Never married (or single) | 167 (6·2%) | 3 (4·8%) | 762 (42·3%) | 28 (45·9%) | 22 (2·0%) | 0 (0·0%) | 951 (17·0%) | 31 (16·8%) | 1·01(0·68 -1·48) | 0·977 |
| Married/ Co-habiting | 2471 (92·1%) | 56 (90·3%) | 1028 (57·1%) | 32 (52·5%) | 1099 (97·6%) | 61 (100·0%) | 4598 (82·0%) | 149 (81·0%) | 1(Ref) |  |
| Separated/Divorced | 35 (1·3%) | 2 (3·2%) | 6 (0·3%) | 1 (1·6%) | 5 (0·4%) | 0 (0·0%) | 46 (0·8%) | 3 (1·6%) | 1·95(0·62 -6·12) | 0·252 |
| Missing | 9 (0·3%) | 1 (1·6%) | 4 (0·2%) | 0 (0·0%) | 0 (0·0%) | 0 (0·0%) | 13 (0·2%) | 1 (0·5%) |  |  |
| **Occupation** |  |  |  |  |  |  |  |  |  |  |
| Housewife | 1458 (54·4%) | 37 (59·7%) | 1300 (72·2%) | 46 (75·4%) | 990 (87·9%) | 52 (85·2%) | 3748 (66·8%) | 135 (73·4%) | 1(Ref) |  |
| Student/Professional | 845 (31·5%) | 18 (29·0%) | 464 (25·8%) | 14 (23·0%) | 81 (7·2%) | 6 (9·8%) | 1390 (24·8%) | 38 (20·7%) | 0·77(0·53 -1·1) | 0·145* |
| Other | 372 (13·9%) | 6 (9·7%) | 35 (1·9%) | 1 (1·6%) | 55 (4·9%) | 3 (4·9%) | 462 (8·2%) | 10 (5·4%) | 0·61(0·32 -1·16) | 0·131* |
| Missing | 7 (0·3%) | 1 (1·6%) | 1 (0·1%) | 0 (0·0%) | 0 (0·0%) | 0 (0·0%) | 8 (0·1%) | 1 (0·5%) |  |  |
| **% likelihood below the poverty line, median (IQR)** | 13·9 (4·6, 30·0) | 17·8 (4·6, 46·4) | 21·4 (7·2, 31·7) | 21·4 (7·2, 31·7) | 46·9 (27·9, 58·6) | 51·3 (34·8, 56·0) | 21·4 (6·1, 42·3) | 31·7 (8·5, 51·3) | 1·01(1·01 -1·02) | <0·001** |
| **Village - rural and urban index** |  |  |  |  |  |  |  |  |  |  |
| Urban/ Peri Urban | 2367 (88·3%) | 42 (67·7%) | 1047 (58·2%) | 38 (62·3%) | 427 (37·8%) | 19 (31·2%) | 3841 (68·5%) | 99 (53·8%) | 1(Ref) |  |
| Rural | 199 (7·4%) | 4 (6·5%) | 753 (41·8%) | 23 (37·7%) | 678 (60·2%) | 39 (63·9%) | 1630 (29·1%) | 66 (35·9%) | 1·54(1·13 -2·11) | 0·006** |
| Missing | 116 (4·3%) | 16 (25·8%) | 0 (0·0%) | 0 (0·0%) | 21 (1·9%) | 3 (4·9%) | 137 (2·4%) | 19 (10·3%) |  |  |
| **INTERMEDIATE FACTORS** |  |  |  |  |  |  |  |  |  |  |
| **Distance to recruiting clinic (km), median (IQR)** | 2·2 (1·8, 6·3) | 2·9 (1·8, 6·4) | 2·0 (1·0, 5·5) | 2·0 (1·0, 4·3) | 1·8 (1·3, 6·4) | 2·5 (1·3, 6·4) | 2·1 (1·3, 6·3) | 2·0 (1·3, 6·2) | 1·00(0·99 -1·00) | 0·919 |
| **Household sanitation access** |  |  |  |  |  |  |  |  |  |  |
| Access to improved sanitation | 2336 (87·1%) | 47 (75·8%) | 1444 (80·2%) | 45 (73·8%) | 687 (61·0%) | 40 (65·6%) | 4467 (79·7%) | 132 (71·7%) | 1(Ref) |  |
| No access to improved sanitation | 336 (12·5%) | 14 (22·6%) | 343 (19·1%) | 15 (24·6%) | 436 (38·7%) | 21 (34·4%) | 1115 (19·9%) | 50 (27·2%) | 1·50(1·08 -2·07) | 0·015** |
| Missing | 10 (0·4%) | 1 (1·6%) | 13 (0·7%) | 1 (1·6%) | 3 (0·3%) | 0 (0·0%) | 26 (0·5%) | 2 (1·1%) |  |  |
| **Household water access** |  |  |  |  |  |  |  |  |  |  |
| Improved/At least limited | 2543 (94·8%) | 54 (87·1%) | 1594 (88·6%) | 55 (90·2%) | 1006 (89·3%) | 53 (86·9%) | 5143 (91·7%) | 162 (88·0%) | 1(Ref) |  |
| Unimproved | 86 (3·2%) | 7 (11·3%) | 187 (10·4%) | 6 (9·8%) | 36 (3·2%) | 3 (4·9%) | 309 (5·5%) | 16 (8·7%) | 1·61(0·96 -2·69) | 0·068* |
| Missing | 53 (2·0%) | 1 (1·6%) | 19 (1·1%) | 0 (0·0%) | 84 (7·5%) | 5 (8·2%) | 156 (2·8%) | 6 (3·3%) |  |  |
| **Household fuel source for cooking** |  |  |  |  |  |  |  |  |  |  |
| Biomass | 743 (27·7%) | 26 (41·9%) | 536 (29·8%) | 20 (32·8%) | 877 (77·9%) | 48 (78·7%) | 2156 (38·4%) | 94 (51·1%) | 2·59 (1·50 – 4·47) | 0·001** |
| Coal | 1186 (44·2%) | 28 (45·2%) | 1087 (60·4%) | 34 (55·7%) | 239 (21·2%) | 12 (19·7%) | 2512 (44·8%) | 74 (40·2%) | 1·78 (1·02 – 3·09) | 0·043** |
| Other (Battery, Electric, Gas, Kerosene) | 744 (27·7%) | 7 (11·3%) | 164 (9·1%) | 7(11·5%) | 8 (0·7%) | 1 (1·6%) | 916 (16·3%) | 15(8·2%) | 1 (Ref) |  |
| Unknown/Missing | 8 (0·3%) | 1 (1·6%) | 2 (0·1%) | 0 (0·0%) | 2 (0·2%) | 0 (0·0%) | 24 (0·4%) | 1 (0·5%) |  |  |
| **Air exposure^a^** |  |  |  |  |  |  |  |  |  |  |
| High: >36 ug/m3 | 913 (34·0%) | 31 (50·0%) | 39 (2·2%) | 1 (1·6%) | 245 (21·8%) | 13 (21·3%) | 1197 (21·8%) | 45 (24·5%) | 0·96 (0·57 – 1·63) | 0·891 |
| Moderate: >=15 and < 36 ug/m3 | 1608 (60·0%) | 28 (45·2%) | 1573 (87·4%) | 53 (86·9%) | 712 (63·2%) | 37 (60·7%) | 3893 (69·4%) | 118 (64·2%) | 0·78 (0·45 – 1·26) | 0·311 |
| Low: <15 ug/m3 | 155 (5·8%) | 2 (3·2%) | 188 (10·4%) | 7 (11·5%) | 169 (15·0%) | 11 (18·0%) | 512 (9·1%) | 20 (10·9%) | 1 (Ref) |  |
| Missing | 6 (0·2%) | 1 (1·6%) | 0 (0·0%) | 0 (0·0%) | 0 (0·0%) | 0 (0·0%) | 0 (0·0%) | 1 (0·5%) |  |  |
| **Conception season** |  |  |  |  |  |  |  |  |  |  |
| Dry | 621 (23·2%) | 7 (11·3%) | 953 (52·9%) | 38 (62·3%) | 685 (60·8%) | 41 (67·2%) | 2259 (40·3%) | 86 (46·7%) | 1(Ref) |  |
| Wet | 1922 (71·7%) | 35 (56·5%) | 842 (46·8%) | 23 (37·7%) | 403 (35·8%) | 17 (27·9%) | 3167 (56·5%) | 75 (40·8%) | 0·63(0·46 -0·86) | 0·004** |
| Missing | 139 (5·2%) | 20 (32·3%) | 5 (0·3%) | 0 (0·0%) | 38 (3·4%) | 3 (4·9%) | 182 (3·2%) | 23 (12·5%) |  |  |
| **Delivery season** |  |  |  |  |  |  |  |  |  |  |
| Dry | 643 (24·0%) | 14 (22·6%) | 994 (55·2%) | 27 (44·3%) | 675 (59·9%) | 35 (57·4%) | 2312 (41·2%) | 76 (41·3%) | 1(Ref) |  |
| Wet | 1908 (71·1%) | 28 (45·2%) | 804 (44·7%) | 34 (55·7%) | 417 (37·0%) | 23 (37·7%) | 3129 (55·8%) | 85 (46·2%) | 0·83(0·61 -1·13) | 0·241 |
| Missing | 131 (4·9%) | 20 (32·3%) | 2 (0·1%) | 0 (0·0%) | 34 (3·0%) | 3 (4·9%) | 167 (3·0%) | 23 (12·5%) |  |  |
| **Maternal Age: median (IQR)** | 27·0 (23·0, 31·0) | 27·5 (23·0, 32·0) | 23·0 (19·0, 29·0) | 25·0 (19·0, 30·0) | 26·0 (22·0, 31·0) | 26·0 (24·0, 32·0) | 26·0 (21·0, 31·0) | 26·0 (22·5, 32·0) | 1·02(1 -1·04) | 0·072* |
| **Maternal Age (categorised)** |  |  |  |  |  |  |  |  |  |  |
| 15-19 | 182 (6·8%) | 5 (8·1%) | 474 (26·3%) | 17 (27·9%) | 141 (12·5%) | 6 (9·8%) | 797 (14·2%) | 28 (15·2%) | 1·39(0·86 -2·25) | 0·179* |
| 20-24 | 808 (30·1%) | 18 (29·0%) | 534 (29·7%) | 11 (18·0%) | 297 (26·4%) | 12 (19·7%) | 1639 (29·2%) | 41 (22·3%) | 1(Ref) |  |
| 25-29 | 792 (29·5%) | 14 (22·6%) | 388 (21·6%) | 16 (26·2%) | 330 (29·3%) | 18 (29·5%) | 1510 (26·9%) | 48 (26·1%) | 1·26(0·83 -1·92) | 0·273 |
| 30-34 | 567 (21·1%) | 14 (22·6%) | 234 (13·0%) | 9 (14·8%) | 205 (18·2%) | 13 (21·3%) | 1006 (17·9%) | 36 (19·6%) | 1·42(0·9 -2·22) | 0·128* |
| 35+ | 333 (12·4%) | 11 (17·7%) | 170 (9·4%) | 8 (13·1%) | 149 (13·2%) | 12 (19·7%) | 652 (11·6%) | 31 (16·8%) | 1·86(1·17 -2·97) | 0·009* |
| Missing | 0 (0·0%) | 0 (0·0%) | 0 (0·0%) | 0 (0·0%) | 4 (0·4%) | 0 (0·0%) | 4 (0·1%) | 0 (0·0%) |  |  |
| **Maternal BMI at recruitment, median(IQR)** | 24·2 (21·6, 28·0) | 24·1 (21·3, 28·9) | 24·4 (22·1, 27·1) | 24·5 (22·4, 28·2) | 22·1 (19·9, 24·9) | 21·8 (19·1, 24·0) | 23·9 (21·4, 27·1) | 23·3 (20·9, 27·3) | 0·98(0·95 -1·02) | 0·332 |
| **Maternal BMI at recruitment, categorized** |  |  |  |  |  |  |  |  |  |  |
| <18·5 | 103 (3·8%) | 4 (6·5%) | 33 (1·8%) | 2 (3·3%) | 141 (12·5%) | 11 (18·0%) | 277 (4·9%) | 17 (9·2%) | 1·89(1·13 -3·16) | 0·016** |
| 18·5-24·9 | 1403 (52·3%) | 29 (46·8%) | 980 (54·4%) | 30 (49·2%) | 716 (63·6%) | 39 (63·9%) | 3099 (55·3%) | 98 (53·3%) | 1(Ref) |  |
| 25-29·9 | 710 (26·5%) | 16 (25·8%) | 589 (32·7%) | 23 (37·7%) | 195 (17·3%) | 8 (13·1%) | 1494 (26·6%) | 47 (25·5%) | 0·99(0·7 -1·41) | 0·977 |
| ≥30 | 444 (16·6%) | 11 (17·7%) | 198 (11·0%) | 6 (9·8%) | 73 (6·5%) | 3 (4·9%) | 715 (12·7%) | 20 (10·9%) | 0·89(0·55 -1·44) | 0·627 |
| Missing | 22 (0·8%) | 2 (3·2%) |  |  | 1 (0·1%) | 0 (0·0%) | 23 (0·4%) | 2 (1·1%) |  |  |
| **MUAC, median (IQR)** | 26·5 (24·0, 29·5) | 25·6 (23·3, 30·0) | 27·5 (25·6, 30·0) | 28·0 (25·7, 30·4) | 26·3 (24·2, 28·8) | 26·7 (24·6, 29·0) | 26·8 (24·5, 29·5) | 26·9 (24·6, 30·0) | 1·01(0·97 -1·04) | 0·709 |
| **MUAC categorised** |  |  |  |  |  |  |  |  |  |  |
| <23cm – underweight | 370 (13·8%) | 12 (19·4%) | 49 (2·7%) | 2 (3·3%) | 121 (10·7%) | 4 (6·6%) | 540 (9·6%) | 18 (9·8%) | 1·06(0·62 -1·78) | 0·84 |
| 23 - <26·5cm – normal | 938 (35·0%) | 22 (35·5%) | 584 (32·4%) | 17 (27·9%) | 445 (39·5%) | 23 (37·7%) | 1967 (35·1%) | 62 (33·7%) | 1(Ref) |  |
| 26·5 - <30cm - overweight | 724 (27·0%) | 9 (14·5%) | 691 (38·4%) | 23 (37·7%) | 327 (29·0%) | 21 (34·4%) | 1742 (31·1%) | 53 (28·8%) | 0·97(0·67 -1·39) | 0·855 |
| >=30cm – obese | 588 (21·9%) | 15 (24·2%) | 464 (25·8%) | 19 (31·1%) | 208 (18·5%) | 12 (19·7%) | 1260 (22·5%) | 46 (25·0%) | 1·15(0·79 -1·69) | 0·465 |
| Missing | 62 (2·3%) | 4 (6·5%) | 12 (0·7%) | 0 (0·0%) | 25 (2·2%) | 1 (1·6%) | 99 (1·8%) | 5 (2·7%) |  |  |
| **HIV status** |  |  |  |  |  |  |  |  |  |  |
| Negative | 2600 (96·9%) | 61 (98·4%) | 1583 (87·9%) | 56 (91·8%) | 1105 (98·1%) | 61 (100·0%) | 5288 (94·3%) | 178 (96·7%) | 1(Ref) |  |
| Positive | 82 (3·1%) | 1 (1·6%) | 217 (12·1%) | 5 (8·2%) | 21 (1·9%) | 0 (0·0%) | 320 (5·7%) | 6 (3·3%) | 0·57(0·25 -1·27) | 0·169* |
| Missing |  |  |  |  |  |  |  |  |  |  |
| **Female Genital Mutilation** |  |  |  |  |  |  |  |  |  |  |
| Absent | 2026 (75·5%) | 47 (75·8%) | 1716 (95·3%) | 54 (88·5%) | 264 (23·4%) | 11 (18·0%) | 4006 (71·4%) | 112 (60·9%) | 1(Ref) |  |
| Present | 7 (0·3%) | 0 (0·0%) | 8 (0·4%) | 0 (0·0%) | 424 (37·7%) | 13 (21·3%) | 439 (7·8%) | 13 (7·1%) | 1·06(0·6 -1·88) | 0·849 |
| Missing | 649 (24·2%) | 15 (24·2%) | 76 (4·2%) | 7 (11·5%) | 438 (38·9%) | 37 (60·7%) | 1163 (20·7%) | 59 (32·1%) |  |  |
| **Parity** |  |  |  |  |  |  |  |  |  |  |
| 1 | 748 (27·9%) | 17 (27·4%) | 697 (38·7%) | 23 (37·7%) | 207 (18·4%) | 10 (16·4%) | 1652 (29·5%) | 50 (27·2%) | 1·04 (0·73 – 1·47) | 0·842 |
| 2-4 | 1542 (57·5%) | 33 (53·2%) | 944 (52·4%) | 31 (50·8%) | 495 (44·0%) | 23 (37·7%) | 2981 (53·2%) | 87 (47·3%) | 1(Ref) |  |
| 5+ | 392 (14·6%) | 12 (19·4%) | 159 (8·8%) | 7 (11·5%) | 424 (37·7%) | 28 (45·9%) | 975 (17·4%) | 47 (25·5%) | 1·62 (1·14 -2·31) | 0·008** |
| Missing |  |  |  |  |  |  |  |  |  |  |
| **History of previous stillbirth** |  |  |  |  |  |  |  |  |  |  |
| No | 2491 (92·9%) | 44 (71·0%) | 1661 (92·3%) | 54 (88·5%) | 991 (88·0%) | 42 (68·9%) | 5143 (91·7%) | 140 (76·1%) | 1(Ref) |  |
| Yes | 179 (6·7%) | 17 (27·4%) | 95 (5·3%) | 6 (9·8%) | 135 (12·0%) | 19 (31·1%) | 409 (7·3%) | 42 (22·8%) | 3·51(2·49 -4·96) | <0·001** |
| Missing | 12 (0·4%) | 1 (1·6%) | 44 (2·4%) | 1 (1·6%) | 0 (0·0%) | 0 (0·0%) | 56 (1·0%) | 2 (1·1%) |  |  |
| **History of previous Caesarean section** |  |  |  |  |  |  |  |  |  |  |
| No | 2436 (90·8%) | 59 (95·2%) | 1726 (95·9%) | 55 (90·2%) | 1069 (94·9%) | 56 (91·8%) | 5231 (93·3%) | 170 (92·4%) | 1(Ref) |  |
| Yes | 246 (9·2%) | 3 (4·8%) | 74 (4·1%) | 6 (9·8%) | 57 (5·1%) | 5 (8·2%) | 377 (6·7%) | 14 (7·6%) | 1·14(0·66 -1·96) | 0·643 |
| **PROXIMAL FACTORS** |  |  |  |  |  |  |  |  |  |  |
| **Gestation Age at recruitment (Weeks): Median(Q1-Q3)^a^** | 21·7 (16·7, 26·4) | 20·3 (16·9, 29·4) | 19·7 (15·8, 23·6) | 18·1 (14·9, 22·0) | 21·9 (16·0, 27·4) | 17·3 (12·1, 22·6) | 20·9 (16·3, 25·4) | 19·0 (14·1, 25·0) | 0·98(0·96 -1) | 0·028** |
| Missing GA | 21 (0·8%) | 3 (4·8%) | 0 (0·0%) | 0 (0·0%) | 4 (0·4%) | 0 (0·0%) | 25 (0·4%) | 3 (1·6%) |  |  |
| **Tobacco use** |  |  |  |  |  |  |  |  |  |  |
| No | 2658 (99·1%) | 61 (98·4%) | 1794 (99·7%) | 61 (100·0%) | 1085 (96·4%) | 57 (93·4%) | 5537 (98·7%) | 179 (97·3%) | 1(Ref) |  |
| Yes | 9 (0·3%) | 0 (0·0%) | 1 (0·1%) | 0 (0·0%) | 37 (3·3%) | 4 (6·6%) | 47 (0·8%) | 4 (2·2%) | 2·50(0·93 -6·75) | 0·069* |
| Missing | 15 (0·6%) | 1 (1·6%) | 5 (0·3%) | 0 (0·0%) | 4 (0·4%) | 0 (0·0%) | 24 (0·4%) | 1 (0·5%) |  |  |
| **Delivery location** |  |  |  |  |  |  |  |  |  |  |
| PHC/Hospital | 2429 (90·6%) | 56 (90·3%) | 1796 (99·8%) | 55 (90·2%) | 982 (87·2%) | 41 (67·2%) | 5207 (92·8%) | 152 (82·6%) | 1(Ref) |  |
| Outside health facility (home or enroute) | 195 (7·3%) | 2 (3·2%) | 3 (0·2%) | 6 (9·8%) | 128 (11·4%) | 17 (27·9%) | 326 (5·8%) | 25 (13·6%) | 2·51(1·65 -3·83) | <0·001** |
| Missing | 58 (2·2%) | 4 (6·5%) | 1 (0·1%) | 0 (0·0%) | 16 (1·4%) | 3 (4·9%) | 75 (1·3%) | 7 (3·8%) |  |  |
| **Mode of delivery** |  |  |  |  |  |  |  |  |  |  |
| Unassisted vaginal/Cephalic | 2226 (83·0%) | 51 (82·3%) | 1357 (75·4%) | 37 (60·7%) | 1027 (91·2%) | 53 (86·9%) | 4610 (82·2%) | 141 (76·6%) | 1(Ref) |  |
| Vaginal breech | 11 (0·4%) | 1 (1·6%) | 122 (6·8%) | 7 (11·5%) | 4 (0·4%) | 1 (1·6%) | 137 (2·4%) | 9 (4·9%) | 2·08(1·06 -4·08) | 0·033** |
| Operative vaginal | 1 (<1%) | 0 (0·0%) | 133 (7·4%) | 5 (8·2%) | 13 (1·2%) | 0 (0·0%) | 147 (2·6%) | 5 (2·7%) | 1·11(0·45 -2·71) | 0·821 |
| Caesarean section | 413 (15·4%) | 6 (9·7%) | 182 (10·1%) | 12 (19·7%) | 33 (2·9%) | 1 (1·6%) | 628 (11·2%) | 19 (10·3%) | 0·99(0·61 -1·60) | 0·966 |
| Missing | 31 (1·2%) | 4 (6·5%) | 6 (0·3%) | 0 (0·0%) | 49 (4·4%) | 6 (9·8%) | 86 (1·5%) | 10 (5·4%) |  |  |
| **American Heart Association category for blood pressure** |  |  |  |  |  |  |  |  |  |  |
| No hypertension^b^ | 2362 (88·1%) | 46 (74·2%) | 1616 (89·8%) | 48 (78·7%) | 1023 (90·9%) | 48 (78·7%) | 5001 (89·2%) | 142 (77·2%) | 1(Ref) |  |
| Stage 1 Hypertension | 176 (6·6%) | 11 (17·7%) | 156 (8·7%) | 7 (11·5%) | 76 (6·7%) | 6 (9·8%) | 408 (7·3%) | 24 (13·0%) | 2·01(1·31 -3·1) | 0·002** |
| Stage 2 Hypertension | 138 (5·1%) | 5 (8·1%) | 28 (1·6%) | 6 (9·8%) | 26 (2·3%) | 7 (11·5%) | 192 (3·4%) | 18 (9·8%) | 3·10(1·90 -5·07) | <0·001** |
| Missing | 6 (0·2%) | 0 (0·0%) | 0 (0·0%) | 0 (0·0%) | 1 (0·1%) | 0 (0·0%) | 7 (0·1%) | 0 (0·0%) |  |  |
| **Preeclampsia** |  |  |  |  |  |  |  |  |  |  |
| No | 2447 (91·2%) | 52 (83·9%) | 1753 (97·4%) | 54 (88·5%) | 962 (85·4%) | 47 (77·0%) | 5162 (92·0%) | 153 (83·2%) | 1(Ref) |  |
| Yes | 229 (8·5%) | 10 (16·1%) | 47 (2·6%) | 7 (11·5%) | 163 (14·5%) | 14 (23·0%) | 439 (7·8%) | 31 (16·8%) | 2·29(1·56 -3·37) | <0·001** |
| Missing | 6 (0·2%) | 0 (0·0%) |  |  | 1 (0·1%) | 0 (0·0%) | 7 (0·1%) | 0 (0·0%) |  |  |
| **Antepartum Haemorrhage** |  |  |  |  |  |  |  |  |  |  |
| No | 2609 (97·3%) | 57 (91·9%) | 1630 (90·6%) | 51 (83·6%) | 1081 (96·0%) | 52 (85·2%) | 5320 (94·9%) | 160 (87·0%) | 1(Ref) |  |
| Yes | 73 (2·7%) | 5 (8·1%) | 170 (9·4%) | 10 (16·4%) | 45 (4·0%) | 9 (14·8%) | 288 (5·1%) | 24 (13·0%) | 2·64(1·72 -4·05) | <0·001** |
| **Placental Abruption** |  |  |  |  |  |  |  |  |  |  |
| No | 2674 (99·7%) | 61 (98·4%) | 1797 (99·8%) | 60 (98·4%) | 1122 (99·6%) | 58 (95·1%) | 5593 (99·7%) | 179 (97·3%) | 1(Ref) |  |
| Yes | 8 (0·3%) | 1 (1·6%) | 3 (0·2%) | 1 (1·6%) | 4 (0·4%) | 3 (4·9%) | 15 (0·3%) | 5 (2·7%) | 8·06(3·32 -19·6) | <0·001** |
| **Size**  **for gestational age at birth** |  |  |  |  |  |  |  |  |  |  |
| Small for Gestational Age (<10th centile) | 437 (16·3%) | 13 (21·0%) | 327 (18·2%) | 18 (29·5%) | 188 (17·7%) | 8 (13·1%) | 952 (17·0%) | 39 (21·2%) | 2·03(1·38 -3) | <0·001** |
| Appropriate for Gestational Age (10th to 90th centile) | 1661 (61·9%) | 29 (46·8%) | 1342 (74·6%) | 26 (42·6%) | 641 (56·9%) | 17 (27·9%) | 3644 (65·0%) | 72 (39·1%) | 1(Ref) |  |
| Large for Gestational Age (>90th centile) | 175 (6·5%) | 2 (3·2%) | 95 (5·3%) | 1 (1·6%) | 68 (6·0%) | 1 (1·6%) | 338 (6·0%) | 4 (2·2%) | 0·6(0·22 -1·65) | 0·326 |
| Missing | 409 (15·2%) | 18 (29·0%) | 36 (2·0%) | 16 (26·2%) | 229 (20·3%) | 35 (57·4%) | 674 (12·0%) | 69 (37·5%) |  |  |
| **Birth plurality** |  |  |  |  |  |  |  |  |  |  |
| Singleton | 2584 (96·3%) | 54 (87·1%) | 1764 (98%) | 61 (100%) | 1071 (95·1%) | 56 (91·8%) | 5419 (96·6%) | 171 (92·9%) | 1(Ref) |  |
| Multiple^c^ | 98 (3·7%) | 8 (12·9%) | 36 (2%) | 0 (0%) | 55 (4·9%) | 5 (8·2%) | 189 (3·4%) | 13 (7·1%) | 2·10 (1·20, 3·70) | 0·010 |
| Missing | 0 (0%) | 0 (0%) | 0 (0%) | 0 (0%) | 0 (0%) | 0 (0%) | 0 (0%) | 0 (0%) |  |  |
| **Sex of baby** |  |  |  |  |  |  |  |  |  |  |
| Male | 1370 (51·1%) | 28 (45·2%) | 913 (50·7%) | 35 (57·4%) | 552 (49·0%) | 18 (29·5%) | 2835 (50·6%) | 81 (44·0%) | 1·25(0·9 -1·74) | 0·181** |
| Female | 1285 (47·9%) | 24 (38·7%) | 884 (49·1%) | 21 (34·4%) | 566 (50·3%) | 17 (27·9%) | 2735 (48·8%) | 62 (33·7%) | 1(Ref) |  |
| Missing | 27 (1·0%) | 10 (16·1%) | 3 (0·2%) | 5 (8·2%) | 8 (0·7%) | 26 (42·6%) | 38 (0·7%) | 41 (22·3%) |  |  |

*BMI= Body Mass Index, MUAC= mid-upper arm circumference, GA = Gestational age, PHC = Primary health centre, IQR – Interquartile range, * p<0·2, ** p<0·05, ^a^classified according to WHO guidelines, ^b^ Includes women with normal or elevated blood pressure, ^c^ Includes twins only, there were no triplets or higher order births.*

## Table S5·2 Bivariate analysis of determinants of stillbirth

|  | **COMPLETE CASE ANALYSIS** | | | | **IMPUTED DATA** | | | |
| --- | --- | --- | --- | --- | --- | --- | --- | --- |
|  | **TOTAL** | | **BIVARIABLE ANALYSIS USING TOTAL STILLBIRTHS AS OUCTOME COMPARED WITH LIVE BIRTHS** | | **PROPORTIONS**  **(Imputations = 25)** | | **BIVARIABLE ANALYSIS USING TOTAL STILLBIRTHS AS OUCTOME COMPARED WITH LIVE BIRTHS** | |
|  | **livebirth** | **stillbirth** | **RR (95% CI)** | **P-value** | **livebirth** | **stillbirth** | **RR (95% CI)** | **P-value** |
| **DISTAL FACTORS** | N=5608 | N=184 |  |  | Proportion (95% CI) | Proportion (95% CI) |  |  |
| **Maternal Education** |  |  |  |  |  |  |  |  |
| None | 1040 (18·5%) | 58 (31·5%) | 2·60(1·24 -5·45) | 0·011** | 18·5%(17·5% - 19·5%) | 32·1%(25·3% - 39%) | 2·54(1·21 -5·31) | 0·013** |
| Primary/Secondary | 4177 (74·5%) | 117 (63·6%) | 1·34(0·66 -2·75) | 0·421 | 74·6%(73·4% - 75·7%) | 63·9%(56·8% - 70·9%) | 1·31(0·64 -2·68) | 0·457 |
| Higher | 386 (6·9%) | 8 (4·3%) | 1(Ref) |  | 6·9%(6·2% - 7·6%) | 4%(1·1% - 6·9%) | 1(Ref) |  |
| Missing | 5 (0·1%) | 1 (0·5%) |  |  |  |  |  |  |
| **Maternal religion** |  |  |  |  |  |  |  |  |
| Muslim | 2178 (38·8%) | 91 (49·5%) | 1(Ref) |  | 39%(37·7% - 40·3%) | 49·9%(42·6% - 57·3%) | 1(Ref) |  |
| Christian | 3389 (60·4%) | 92 (50·0%) | 0·66(0·49 -0·88) | 0·005** | 61%(59·7% - 62·3%) | 50·1%(42·7% - 57·4%) | 0·66(0·49 -0·88) | 0·005** |
| Missing | 41 (0·7%) | 1 (0·5%) |  |  |  |  |  |  |
| **Marital status** |  |  |  |  |  |  |  |  |
| Never married (or single) | 951 (17·0%) | 31 (16·8%) | 1·01(0·68 -1·48) | 0·977 | 17%(16% - 18%) | 17·2%(11·6% - 22·7%) | 1(0·68 -1·48) | 0·983 |
| Married/ Co-habiting | 4598 (82·0%) | 149 (81·0%) | 1(Ref) |  | 82·1%(81·1% - 83·2%) | 81·1%(75·4% - 86·9%) | 1(Ref) |  |
| Separated/Divorced | 46 (0·8%) | 3 (1·6%) | 1·95(0·62 -6·12) | 0·252 | 0·8%(0·6% - 1·1%) | 1·7%(-0·2% - 3·5%) | 1·94(0·62 -6·08) | 0·257 |
| Missing | 13 (0·2%) | 1 (0·5%) |  |  |  |  |  |  |
| **Occupation** |  |  |  |  |  |  |  |  |
| Housewife | 3748 (66·8%) | 135 (73·4%) | 1(Ref) |  | 67%(65·7% - 68·2%) | 74·9%(68·5% - 81·3%) | 1(Ref) |  |
| Student/Professional | 1390 (24·8%) | 38 (20·7%) | 0·77(0·53 -1·1) | 0·145* | 24·8%(23·7% - 26%) | 20·6%(14·7% - 26·6%) | 0·77(0·54 -1·1) | 0·152* |
| Other | 462 (8·2%) | 10 (5·4%) | 0·61(0·32 -1·16) | 0·131* | 8·2%(7·5% - 8·9%) | 4·5%(1·4% - 7·5%) | 0·61(0·32 -1·17) | 0·136* |
| Missing | 8 (0·1%) | 1 (0·5%) |  |  |  |  |  |  |
| **% likelihood below the poverty line, median (IQR)** | 21·4 (6·1, 42·3) | 31·7 (8·5, 51·3) | 1·01(1·01 -1·02) | <0·001** | 21·4 (6·1, 4·2.3) | 31·7  (8·6, 51·3) | 1·01(1·01 -1·02) | <0·001** |
| **Village - rural and urban index** |  |  |  |  |  |  |  |  |
| Urban/ Peri Urban | 3841 (68·5%) | 99 (53·8%) | 1(Ref) |  | 70·3%(69% - 71·5%) | 60·2%(52·7% - 67·6%) | 1(Ref) |  |
| Rural | 1630 (29·1%) | 66 (35·9%) | 1·54(1·13 -2·11) | 0·006** | 29·7%(28·5% - 31%) | 39·8%(32·4% - 47·3%) | 1·50(1·11 -2·04) | 0·009** |
| Missing | 137 (2·4%) | 19 (10·3%) |  |  |  |  |  |  |
| **INTERMEDIATE FACTORS** |  |  |  |  |  |  |  |  |
| **Distance from clinic at booking, median (IQR)** | 2·1 (1·3, 6·3) | 2·0 (1·3, 6·2) | 1·00(0·99 -1·00) | 0·919 | 2·1 (1·3, 6·7) | 2·1 (1·3, 7·9) | 1·00(0·99 -1·01) | 0·940 |
| **Sanitation** |  |  |  |  |  |  |  |  |
| Access to improved sanitation | 4467 (79·7%) | 132 (71·7%) | 1(Ref) |  | 80·1%(79% - 81·1%) | 72·1%(65·5% - 78·7%) | 1(Ref) |  |
| No access to improved sanitation | 1115 (19·9%) | 50 (27·2%) | 1·50(1·08 -2·07) | 0·015** | 19·9%(18·9% - 21%) | 27·9%(21·3% - 34·5%) | 1·49(1·08 -2·07) | 0·016** |
| Missing | 26 (0·5%) | 2 (1·1%) |  |  |  |  |  |  |
| **Water** |  |  |  |  |  |  |  |  |
| Improved/At least limited | 5143 (91·7%) | 162 (88·0%) | 1(Ref) |  | 94·3%(93·7% - 94·9%) | 90·9%(86·6% - 95·2%) | 1(Ref) |  |
| Unimproved | 309 (5·5%) | 16 (8·7%) | 1·61(0·96 -2·69) | 0·068* | 5·7%(5·1% - 6·3%) | 9·1%(4·8% - 13·4%) | 1·60(0·96 -2·67) | 0·073* |
| Missing | 156 (2·8%) | 6 (3·3%) |  |  |  |  |  |  |
| **Fuel source for cooking** |  |  |  |  |  |  |  |  |
| Biomass | 2156 (38·4%) | 94 (51·1%) | 2·59 (1·50 – 4·47) | 0·001** | 38·7%(37·4% - 39·9%) | 52·2%(44·8% - 59·5%) | 2·56(1·49 -4·41) | 0·001** |
| Coal | 2512 (44·8%) | 74 (40·2%) | 1·78 (1·02 – 3·09) | 0·043** | 45%(43·7% - 46·3%) | 40%(32·8% - 47·2%) | 1·76(1·01 -3·06) | 0·046** |
| Other (Battery, Electric, Gas, Kerosene) | 916 (16·3%) | 15(8·2%) | 1 (Ref) |  | 16·3%(15·3% - 17·3%) | 7·9%(3·9% - 11·8%) | 1(Ref) |  |
| Unknown/Missing | 24 (0·4%) | 1 (0·5%) |  |  |  |  |  |  |
| **Air exposure^a^** |  |  |  |  |  |  |  |  |
| High: >36 ug/m3 | 1197 (21·8%) | 45 (24·5%) | 0·96 (0·57 – 1·63) | 0·891 | 24·7%(18·4% - 30·9%) | 21·4%(20·3% - 22·4%) | 0·97(0·57 -1·64) | 0·908 |
| Moderate: >=15 and < 36 ug/m3 | 3893 (69·4%) | 118 (64·2%) | 0·78 (0·45 – 1·26) | 0·311 | 64·5%(57·5% - 71·4%) | 69·5%(68·3% - 70·7%) | 0·79(0·49 -1·26) | 0·317 |
| Low (< WHO guideline): <15 ug/m3 | 512 (9·1%) | 20 (10·9%) | 1 (Ref) |  | 10·9%(6·4% - 15·4%) | 9·1%(8·4% - 9·9%) | 1(Ref) |  |
| Missing | 0 (0·0%) | 1 (0·5%) |  |  |  |  |  |  |
| **Conception season: data** |  |  |  |  |  |  |  |  |
| Dry | 2259 (40·3%) | 86 (46·7%) | 1(Ref) |  | 41·6%(40·2% - 42·9%) | 51·7%(44·2% - 59·2%) | 1(Ref) |  |
| Wet | 3167 (56·5%) | 75 (40·8%) | 0·63(0·46 -0·86) | 0·004** | 58·4%(57·1% - 59·8%) | 48·3%(40·8% - 55·8%) | 0·68(0·5 -0·92) | 0·011** |
| Missing | 182 (3·2%) | 23 (12·5%) |  |  |  |  |  |  |
| **Delivery season: data** |  |  |  |  |  |  |  |  |
| Dry | 2312 (41·2%) | 76 (41·3%) | 1(Ref) |  | 42·3%(41% - 43·7%) | 46·2%(38·4% - 54%) | 1(Ref) |  |
| Wet | 3129 (55·8%) | 85 (46·2%) | 0·83(0·61 -1·13) | 0·241 | 57·7%(56·3% - 59%) | 53·8%(46% - 61·6%) | 0·87(0·64 -1·18) | 0·367 |
| Missing | 167 (3·0%) | 23 (12·5%) |  |  |  |  |  |  |
| **Maternal Age: median(Q1-Q3)** | 26·0 (21·0, 31·0) | 26·0 (22·5, 32·0) | 1·02(1 -1·04) | 0·072* | 26·0(21·0, 31·0) | 26·0 (23·0, 32·0) | 1·02(1 -1·04) | 0·072* |
| **Maternal Age (categorised)** |  |  |  |  |  |  |  |  |
| 15-19 | 797 (14·2%) | 28 (15·2%) | 1·39(0·86 -2·25) | 0·179* | 14·2%(13·3% - 15·2%) | 15·5%(10·2% - 20·8%) | 1·39(0·86 -2·25) | 0·178* |
| 20-24 | 1639 (29·2%) | 41 (22·3%) | 1(Ref) |  | 29·2%(28% - 30·4%) | 22·7%(16·5% - 28·8%) | 1(Ref) |  |
| 25-29 | 1510 (26·9%) | 48 (26·1%) | 1·26(0·83 -1·92) | 0·273 | 27%(25·9% - 28·2%) | 26%(19·5% - 32·4%) | 1·26(0·83 -1·91) | 0·274 |
| 30-34 | 1006 (17·9%) | 36 (19·6%) | 1·42(0·9 -2·22) | 0·128* | 17·9%(16·9% - 18·9%) | 18·8%(13·1% - 24·5%) | 1·42(0·9 -2·21) | 0·128* |
| 35+ | 652 (11·6%) | 31 (16·8%) | 1·86(1·17 -2·97) | 0·009* | 11·6%(10·8% - 12·5%) | 17·1%(11·6% - 22·7%) | 1·86(1·17 -2·96) | 0·009** |
| Missing | 4 (0·1%) | 0 (0·0%) |  |  |  |  |  |  |
| **Maternal BMI** | 23·9 (21·4, 27·1) | 23·3 (20·9, 27·3) | 0·98(0·95 -1·02) | 0·332 | 23·9 (21·4, 27·1) | 23·3 (20·9, 27·3) | 0·98(0·95 -1·02) | 0·332 |
| **Maternal BMI categorized** |  |  |  |  |  |  |  |  |
| <18·5 | 277 (4·9%) | 17 (9·2%) | 1·89(1·13 -3·16) | 0·016** | 4·9%(4·3% - 5·5%) | 9·4%(5·1% - 13·7%) | 1·88(1·12 -3·14) | 0·017** |
| 18·5-24·9 | 3099 (55·3%) | 98 (53·3%) | 1(Ref) |  | 55·5%(54·2% - 56·8%) | 54·6%(47·3% - 62%) | 1(Ref) |  |
| 25-29·9 | 1494 (26·6%) | 47 (25·5%) | 0·99(0·7 -1·41) | 0·977 | 26·8%(25·6% - 27·9%) | 25·3%(18·8% - 31·7%) | 1(0·71 -1·41) | 0·996 |
| ≥30 | 715 (12·7%) | 20 (10·9%) | 0·89(0·55 -1·44) | 0·627 | 12·8%(12% - 13·7%) | 10·7%(6·1% - 15·3%) | 0·89(0·55 -1·44) | 0·637 |
| Missing | 23 (0·4%) | 2 (1·1%) |  |  |  |  |  |  |
| **Average MUAC, median (IQR)** | 26·8 (24·5, 29·5) | 26·9 (24·6, 30·0) | 1·01(0·97 -1·04) | 0·709 | 26·8 (24·6, 29·5) | 26·9 (24·6, 30·1) | 1·01(0·97 -1·04) | 0·709 |
| **Average MUAC categorised** |  |  |  |  |  |  |  |  |
| <23cm – underweight | 540 (9·6%) | 18 (9·8%) | 1·06(0·62 -1·78) | 0·84 | 9·9%(9·1% - 10·6%) | 10·3%(5·8% - 14·9%) | 1·06(0·63 -1·8) | 0·815 |
| 23 - <26·5cm – normal | 1967 (35·1%) | 62 (33·7%) | 1(Ref) |  | 35·8%(34·5% - 37·1%) | 34·7%(27·6% - 41·7%) | 1(Ref) |  |
| 26·5 - <30cm - overweight | 1742 (31·1%) | 53 (28·8%) | 0·97(0·67 -1·39) | 0·855 | 31·6%(30·3% - 32·8%) | 30·3%(23·4% - 37·1%) | 0·97(0·67 -1·4) | 0·886 |
| >=30cm – obese | 1260 (22·5%) | 46 (25·0%) | 1·15(0·79 -1·69) | 0·465 | 22·8%(21·7% - 23·9%) | 24·8%(18·3% - 31·2%) | 1·15(0·79 -1·68) | 0·473 |
| Missing | 99 (1·8%) | 5 (2·7%) |  |  |  |  |  |  |
| **HIV status** |  |  |  |  |  |  |  |  |
| No | 5288 (94·3%) | 178 (96·7%) | 1(Ref) |  | 94·3%(93·7% - 94·9%) | 96·7%(94·1% - 99·3%) | 1(Ref) |  |
| Yes | 320 (5·7%) | 6 (3·3%) | 0·57(0·25 -1·27) | 0·169* | 5·7%(5·1% - 6·3%) | 3·3%(0·7% - 5·9%) | 0·57(0·25 -1·27) | 0·169* |
| Missing |  |  |  |  |  |  |  |  |
| **Female Genital Mutilation** |  |  |  |  |  |  |  |  |
| No | 4006 (71·4%) | 112 (60·9%) | 1(Ref) |  | 88·4%(87·3% - 89·4%) | 82·7%(75·7% - 89·6%) | 1(Ref) |  |
| Yes | 439 (7·8%) | 13 (7·1%) | 1·06(0·6 -1·88) | 0·849 | 11·6%(10·6% - 12·7%) | 17·3%(10·4% - 24·3%) | 1·53(0·95 -2·46) | 0·081* |
| Missing | 1163 (20·7%) | 59 (32·1%) |  |  |  |  |  |  |
| **Parity** |  |  |  |  |  |  |  |  |
| 1 | 1652 (29·5%) | 50 (27·2%) | 1·04 (0·73 – 1·47) | 0·842 | 29·4%(28·2% - 30·6%) | 27·1%(20·6% - 33·6%) | 1·04(0·73 -1·47) | 0·842 |
| 2-4 | 2981 (53·2%) | 87 (47·3%) | 1(Ref) |  | 53·1%(51·8% - 54·5%) | 47%(39·6% - 54·3%) | 1(Ref) |  |
| 5+ | 975 (17·4%) | 47 (25·5%) | 1·62 (1·14 -2·31) | 0·008** | 17·4%(16·4% - 18·4%) | 26%(19·5% - 32·4%) | 1·62(1·14 -2·31) | 0·008** |
| Missing |  |  |  |  |  |  |  |  |
| **Previous stillbirth** |  |  |  |  |  |  |  |  |
| No | 5143 (91·7%) | 140 (76·1%) | 1(Ref) |  | 92·6%(91·9% - 93·3%) | 76·8%(70·5% - 83%) | 1(Ref) |  |
| Yes | 409 (7·3%) | 42 (22·8%) | 3·51(2·49 -4·96) | <0·001** | 7·4%(6·7% - 8·1%) | 23·2%(17% - 29·5%) | 3·48(2·46 -4·91) | <0·001** |
| Missing | 56 (1·0%) | 2 (1·1%) |  |  |  |  |  |  |
| **Previous Caesarean section** |  |  |  |  |  |  |  |  |
| No | 5231 (93·3%) | 170 (92·4%) | 1(Ref) |  | 93·3%(92·6% - 94%) | 92·3%(88·3% - 96·2%) | 1(Ref) |  |
| Yes | 377 (6·7%) | 14 (7·6%) | 1·14(0·66 -1·96) | 0·643 | 6·7%(6% - 7·4%) | 7·7%(3·8% - 11·7%) | 1·14(0·66 -1·96) | 0·643 |
| **PROXIMAL FACTORS** |  |  |  |  |  |  |  |  |
| **Tobacco use** |  |  |  |  |  |  |  |  |
| No | 5537 (98·7%) | 179 (97·3%) | 1(Ref) |  | 99·2%(98·9% - 99·4%) | 97·8%(95·6% - 99·9%) | 1(Ref) |  |
| Yes | 47 (0·8%) | 4 (2·2%) | 2·50(0·93 -6·75) | 0·069* | 0·8%(0·6% - 1·1%) | 2·2%(0·1% - 4·4%) | 2·49(0·92 -6·7) | 0·071* |
| Missing | 24 (0·4%) | 1 (0·5%) |  |  |  |  |  |  |
| **Delivery location** |  |  |  |  |  |  |  |  |
| PHC/Hospital | 5207 (92·8%) | 152 (82·6%) | 1(Ref) |  | 94·1%(93·4% - 94·7%) | 85·9%(80·7% - 91%) | 1(Ref) |  |
| Outside health facility (home or enroute) | 326 (5·8%) | 25 (13·6%) | 2·51(1·65 -3·83) | <0·001** | 5·9%(5·3% - 6·6%) | 14·1%(9% - 19·3%) | 2·47(1·62 -3·77) | <0·001** |
| Missing | 75 (1·3%) | 7 (3·8%) |  |  |  |  |  |  |
| **Mode of delivery** |  |  |  |  |  |  |  |  |
| Unassisted vaginal/Cephalic | 4610 (82·2%) | 141 (76·6%) | 1(Ref) |  | 83·6%(82·7% - 84·6%) | 81·4%(75·6% - 87·1%) | 1(Ref) |  |
| Operative vaginal | 147 (2·6%) | 5 (2·7%) | 1·11(0·45 -2·71) | 0·821 | 2·6%(2·2% - 3·1%) | 2·8%(0·4% - 5·3%) | 1·11(0·45 -2·7) | 0·827 |
| Vaginal breech | 137 (2·4%) | 9 (4·9%) | 2·08(1·06 -4·08) | 0·033** | 2·5%(2·1% - 2·9%) | 5%(1·8% - 8·3%) | 2·03(1·04 -3·99) | 0·039** |
| Caesarean section | 628 (11·2%) | 19 (10·3%) | 0·99(0·61 -1·60) | 0·966 | 11·2%(10·4% - 12·1%) | 10·8%(6·2% - 15·4%) | 1·01(0·63 -1·62) | 0·98 |
| Missing | 86 (1·5%) | 10 (5·4%) |  |  |  |  |  |  |
| **American Heart Association category for blood pressure** |  |  |  |  |  |  |  |  |
| No hypertension^b^ | 5001 (89·2%) | 142 (77·2%) | 1(Ref) |  | 89·3%(88·5% - 90·1%) | 77·9%(71·8% - 84%) | 1(Ref) |  |
| Stage 1 Hypertension | 408 (7·3%) | 24 (13·0%) | 2·01(1·31 -3·1) | 0·002** | 7·3%(6·6% - 8%) | 12·2%(7·4% - 16·9%) | 2·01(1·31 -3·1) | 0·002** |
| Stage 2 Hypertension | 192 (3·4%) | 18 (9·8%) | 3·10(1·90 -5·07) | <0·001** | 3·4%(2·9% - 3·9%) | 9·9%(5·6% - 14·3%) | 3·1(1·9 -5·07) | <0·001** |
| Missing | 7 (0·1%) | 0 (0·0%) |  |  |  |  |  |  |
| **Preeclampsia** |  |  |  |  |  |  |  |  |
| No | 5162 (92·0%) | 153 (83·2%) | 1(Ref) |  | 92·1%(91·4% - 92·8%) | 82·9%(77·3% - 88·4%) | 1(Ref) |  |
| Yes | 439 (7·8%) | 31 (16·8%) | 2·29(1·56 -3·37) | <0·001** | 7·9%(7·2% - 8·6%) | 17·1%(11·6% - 22·7%) | 2·29(1·56 -3·37) | <0·001** |
| Missing | 7 (0·1%) | 0 (0·0%) |  |  |  |  |  |  |
| **Antepartum Haemorrhage** |  |  |  |  |  |  |  |  |
| No | 5320 (94·9%) | 160 (87·0%) | 1(Ref) |  | 94·9%(94·3% - 95·4%) | 86·7%(81·8% - 91·7%) | 1(Ref) |  |
| Yes | 288 (5·1%) | 24 (13·0%) | 2·64(1·72 -4·05) | <0·001** | 5·1%(4·6% - 5·7%) | 13·3%(8·3% - 18·2%) | 2·63(1·72 -4·05) | <0·001** |
| **Placental Abruption** |  |  |  |  |  |  |  |  |
| No | 5593 (99·7%) | 179 (97·3%) | 1(Ref) |  | 99·7%(99·6% - 99·9%) | 97·2%(94·8% - 99·6%) | 1(Ref) |  |
| Yes | 15 (0·3%) | 5 (2·7%) | 8·06(3·32 -19·6) | <0·001** | 0·3%(0·1% - 0·4%) | 2·8%(0·4% - 5·2%) | 8·06(3·31 -19·6) | <0·001** |
| **Birth plurality** |  |  |  |  |  |  |  |  |
| Singleton | 5419 (96·6%) | 171 (92·9%) | 1(Ref) |  | 96·7%(96·2% - 97·1%) | 92·9%(89·2% - 96·7%) | 1(Ref) |  |
| Multiple | 189 (3·4%) | 13 (7·1%) | 2·10 (1·20, 3·70) | 0·010 | 3·3%(2·9% - 3·8%) | 7·1%(3·3% - 10·8%) | 2·13 (1·21, 3·74) | 0·009 |
| **Size**  **for gestational age at birth** |  |  |  |  |  |  |  |  |
| Small for Gestational Age (<10th centile) | 952 (17·0%) | 39 (21·2%) | 2·03(1·38 -3) | <0·001** | 19·8%(18·6% - 21%) | 26·8%(19·3% - 34·3%) | 1·45(1 -2·1) | 0·050* |
| Appropriate for Gestational Age (10th to 90th centile) | 3644 (65·0%) | 72 (39·1%) | 1(Ref) |  | 73·4%(72·1% - 74·7%) | 66·7%(58·6% - 74·8%) | 1(Ref) |  |
| Large for Gestational Age (>90th centile) | 338 (6·0%) | 4 (2·2%) | 0·6(0·22 -1·65) | 0·326 | 6·8%(6·1% - 7·5%) | 6·5%(1·2% - 11·8%) | 1·03(0·44 -2·42) | 0·940 |
| Missing | 674 (12·0%) | 69 (37·5%) |  |  |  |  |  |  |
| **Sex of baby** |  |  |  |  |  |  |  |  |
| Male | 2835 (50·6%) | 81 (44·0%) | 1·25(0·9 -1·74) | 0·181** | 50·9%(49·6% - 52·2%) | 56·2%(48·3% - 64·1%) | 1·22(0·89 -1·68) | 0·210 |
| Female | 2735 (48·8%) | 62 (33·7%) | 1(Ref) |  | 49·1%(47·8% - 50·4%) | 43·8%(35·9% - 51·7%) | 1(Ref) |  |
| Missing | 38 (0·7%) | 41 (22·3%) |  |  |  |  |  |  |

BMI= Body Mass Index, MUAC= mid-upper arm circumference, PHC = primary health centre * p<0·2, ** p<0·05,

^a^classified according to WHO guidelines, ^b^ Includes women with normal or elevated blood pressure

## Table S5·3 Bivariate analysis of additional factors associated with stillbirth (complete case analyses)

|  | **KENYA** | | **MOZAMBIQUE** | | **THE GAMBIA** | | **TOTAL** | | **Association with stillbirth** | |
| --- | --- | --- | --- | --- | --- | --- | --- | --- | --- | --- |
|  | **livebirth** | **stillbirth** | **livebirth** | **stillbirth** | **livebirth** | **stillbirth** | **livebirth** | **stillbirth** | **RR (95% CI)** | **P-value** |
| **PROXIMAL FACTORS** | **N=2682** | **N=62** | **N=1800** | **N=61** | **N=1126** | **N=61** | **N=5608** | **N=184** |  |  |
| **Maternal religion** |  |  |  |  |  |  |  |  |  |  |
| Muslim | 1036 (38·6%) | 29 (46·8%) | 23 (1·3%) | 2 (3·3%) | 1119 (99·4%) | 60 (98·4%) | 2178 (38·8%) | 91 (49·5%) | 1(Ref) |  |
| Christian | 1630 (60·8%) | 32 (51·6%) | 1752 (97·3%) | 59 (96·7%) | 7 (0·6%) | 1 (1·6%) | 3389 (60·4%) | 92 (50·0%) | 0·66(0·49 -0·88) | 0·005** |
| Other/Missing | 16 (0·6%) | 1 (1·6%) | 54 (1·4%) | 0 (0·0%) | 0 (0·0%) | 0 (0·0%) | 41 (0·7%) | 1 (0·5%) |  |  |
| **Reported sleeping position at recruitment*** |  |  |  |  |  |  |  |  |  |  |
| On back | 118 (4·4%) | 3 (4·8%) | 43 (2·4%) | 2 (3·3%) | 32 (2·8%) | 1 (1·6%) | 193 (3·4%) | 6 (3·3%) | 0·95(0·42 -2·14) | 0·899 |
| On stomach | 47 (1·8%) | 2 (3·2%) | 8 (0·4%) | 0 (0·0%) | 18 (1·6%) | 1 (1·6%) | 73 (1·3%) | 3 (1·6%) | 1·24(0·4 -3·89) | 0·709 |
| On side | 2507 (93·5%) | 56 (90·3%) | 1720 (95·6%) | 59 (96·7%) | 1075 (95·5%) | 59 (96·7%) | 5302 (94·5%) | 174 (94·6%) | 1(Ref) |  |
| Missing | 10 (0·4%) | 1 (1·6%) | 29 (1·6%) | 0 (0·0%) | 1 (0·1%) | 0 (0·0%) | 40 (0·7%) | 1 (0·5%) |  |  |
| **Gestation Age at Delivery (Weeks): Median(Q1-Q3)** | 39·1 (37·3, 40·6) | 35·4 (30·4, 38·0) | 39·6 (38·1, 40·9) | 34·7 (29·4, 38·1) | 39·0 (37·4, 40·4) | 35·1 (28·4, 39·4) | 39·3 (37·7, 40·6) | 35·3 (29·3, 38·3) | 0·86(0·85 -0·88) | <0·001** |
| **Gestation Age at Delivery (categorical)** |  |  |  |  |  |  |  |  |  |  |
| 20 - 27 weeks | 15 (0·6%) | 10 (16·1%) | 5 (0·3%) | 12 (19·7%) | 3 (0·3%) | 14 (23·0%) | 23 (0·4%) | 36 (19·6%) | 42·67(28·19 -64·59) | <0·001** |
| 28 – 31 weeks | 55 (2·1%) | 10 (16·1%) | 22 (1·2%) | 12 (19·7%) | 25 (2·2%) | 10 (16·4%) | 102 (1·8%) | 32 (17·4%) | 16·7(10·86 -25·68) | <0·001** |
| 32 – 36 weeks | 485 (18·1%) | 21 (33·9%) | 208 (11·6%) | 15 (24·6%) | 185 (16·4%) | 13 (21·3%) | 878 (15·7%) | 49 (26·6%) | 3·7(2·53 -5·4) | <0·001** |
| 37 – 41 weeks | 1896 (70·7%) | 19 (30·6%) | 1393 (77·4%) | 20 (32·8%) | 778 (69·1%) | 20 (32·8%) | 4067 (72·5%) | 59 (32·1%) | 1(Ref) |  |
| 42 weeks or more | 178 (6·6%) | 1 (1·6%) | 165 (9·2%) | 2 (3·3%) | 100 (8·9%) | 3 (4·9%) | 443 (7·9%) | 6 (3·3%) | 0·93(0·4 -2·16) | 0·874 |
| Missing | 53 (2·0%) | 1 (1·6%) | 7 (0·4%) | 0 (0·0%) | 35 (3·1%) | 1 (1·6%) | 95 (1·7%) | 2 (1·1%) |  |  |

* Not included in final model as only available at recruitment and not in the 3^rd^ trimester

## Table S5·4 Multivariable analysis of risk factors for stillbirths ≥20 weeks (Imputed dataset analysis)

|  | **MODEL 1: ADJUSTED FOR DISTAL FACTORS** | | **MODEL 2: ADJUSTED FOR DISTAL AND INTERMEDIATE FACTOR** | | **MODEL 3: ADJUSTED FOR DISTAL, INTERMEDIATE AND PROXIMAL FACTORS** | |
| --- | --- | --- | --- | --- | --- | --- |
|  | **aRR (95% CI)** | **P-value** | **aRR (95% CI)** | **P-value** | **aRR (95% CI)** | **P-value** |
| **Maternal Education** |  |  |  |  |  |  |
| None | 1·71(0·78 - 3·76) | 0·180 | 1·54 (0·71 -3·38) | 0·278 | 1·48(0·67 -3·24) | 0·331 |
| Primary/ Secondary | 1·12(0·54 -2·3) | 0·767 | 1·12(0·55 -2·32) | 0·747 | 1·16(0·56 -2·39) | 0·695 |
| Higher | 1(Ref) |  | 1(Ref) |  | 1(Ref) |  |
| **Village - rural and urban index** |  |  |  |  |  |  |
| Urban / Peri Urban | 1(Ref) |  | 1(Ref) |  | 1(Ref) |  |
| Rural | 1·21(0·87 -1·67) | 0·253 | 1·13(0·82 -1·58) | 0·443 | 1·10(0·79 -1·54) | 0·576 |
| **% likelihood below the poverty line, median (IQR)** | 1·01(1 -1·02) | 0·018 | 1·01(1 -1·01) | 0·063 | 1·01(0·99 -1·01) | 0·128 |
| **History of previous stillbirth** |  |  |  |  |  |  |
| No |  |  | 1(Ref) |  | 1(Ref) |  |
| Yes |  |  | 3·08(2·17 -4·37) | <0·001** | 2·76(1·93 -3·94) | <0·001** |
| **Household water access** |  |  |  |  |  |  |
| Improved/At least limited |  |  | 1(Ref) |  | 1(Ref) |  |
| Unimproved |  |  | 1·38(0·9 -2·13) | 0·235 | 1·43(0·84 -2·41) | 0·184 |
| **Conception season: data** |  |  |  |  |  |  |
| Dry |  |  | 1(Ref) |  | 1(Ref) |  |
| Wet |  |  | 0·75(0·56 -1·02) | 0·067 | 0·75(0·55 -1·02) | 0·067 |
| **Tobacco use during pregnancy** |  |  |  |  |  |  |
| No |  |  |  |  | 1(Ref) |  |
| Yes |  |  |  |  | 2·19(0·81 -5·99) | 0·124 |
| **American Heart Association category for blood pressure** |  |  |  |  |  |  |
| No hypertension^a^ |  |  |  |  | 1(Ref) |  |
| Stage 1 Hypertension |  |  |  |  | 1·85(1·19 -2·86) | 0·006 |
| Stage 2 Hypertension |  |  |  |  | 2·09(1·14 -3·83) | 0·018 |
| **Preeclampsia** |  |  |  |  |  |  |
| No |  |  |  |  | 1(Ref) |  |
| Yes |  |  |  |  | 1·59(0·91 -2·45) | 0·109 |
| **Antepartum Haemorrhage** |  |  |  |  |  |  |
| No |  |  |  |  | 1(Ref) |  |
| Yes |  |  |  |  | 2·53(1·59 -4·02) | <0·001** |
| **Placental Abruption** |  |  |  |  |  |  |
| No |  |  |  |  | 1(Ref) |  |
| Yes |  |  |  |  | 2·68(0·99 -7·28) | 0·053 |
| **Delivery location** |  |  |  |  |  |  |
| PHC/Hospital |  |  |  |  | 1(Ref) |  |
| Outside health facility (home or enroute) |  |  |  |  | 2. 32(1·49-3·62) | <0·001** |
| **Size for gestational age at birth** |  |  |  |  |  |  |
| Small for Gestational Age (<10th centile) |  |  |  |  | 1·33(0·92 -1·92) | 0·131 |
| Appropriate for Gestational Age (10th to 90th centile) |  |  |  |  | 1(Ref) |  |
| Large for Gestational Age (>90th centile) |  |  |  |  | 0·97 (0·42 -2·25) | 0·945 |
| Akaike's information criterion (AIC)^#^ | **1622·33** |  | **1590·72** |  | **1551·38** |  |
| Bayesian information criterion (BIC)^#^ | **1655·65** |  | **1644·04** |  | **1664·67** |  |

All analyses based on 5608 livebirths and 184 stillbirths. BMI= Body Mass Index, PHC = primary health centre, aRR= adjusted relative risk, adjusted for other variables in the model

^a^ Includes women with normal or elevated blood pressure, ^#^ AIC and BIC are a proxy obtained from the 1st sample of the 25 multiple imputed samples

## Table S5·5 Sensitivity analysis: Multivariable model (Model 3) with and without country as a covariate (complete-case analysis)

|  | **MODEL 3: PRIMARY** (without country) | | **MODEL 3: SENSITIVITY** (with country) | |
| --- | --- | --- | --- | --- |
|  | **Livebirth=4702; stillbirths=101** | | **Livebirth=4702; stillbirths=101** | |
|  | **RR (95% CI)** | **P-value** | **RR (95% CI)** | **P-value** |
| **Maternal Education** |  |  |  |  |
| None | 1·70(0·54 -5·31) | 0·363 | 1·60(0·50 -5·12) | 0·425 |
| Primary | 1·74(0·61 -5·01) | 0·304 | 1·63(0·56 -4·73) | 0·372 |
| Secondary | 1·23(0·43 -3·61) | 0·697 | 1·04(0·35 -3·10) | 0·943 |
| Higher | 1(Ref) |  | 1(Ref) |  |
| **Village - rural and urban index** |  |  |  |  |
| Urban / Peri Urban | 1(Ref) |  | 1(Ref) |  |
| Rural | 1·03(0·66 -1·62) | 0·865 | 0·93(0·58 -1·47) | 0·743 |
| **% likelihood below the poverty line, median (IQR)** | 1·01(1 -1·02) | 0·086 | 1·01(1·00 -1·02) | 0·133 |
| **Previous stillbirth** |  |  |  |  |
| No | 1(Ref) |  | 1(Ref) |  |
| Yes | 3·18(1·85 -5·46) | <0·001** | 3·17(1·85 -5·44) | <0·001** |
| **Maternal BMI (categorised)** |  |  |  |  |
| <18·5 | 1·39(0·62 -3·15) | 0·427 | 1·43(0·63 -3·27) | 0·394 |
| 18·5-24·9 | 1(Ref) |  | 1(Ref) |  |
| 25-29·9 | 1·40(0·88 -2·24) | 0·155 | 1·36(0·85 -2·17) | 0·195 |
| ≥30 | 1·14(0·59 -2·20) | 0·689 | 1·13(0·59 -2·19) | 0·706 |
| **Maternal Age (categorised)** |  |  |  |  |
| 15-19 | 1·60(0·81 -3·14) | 0·173 | 1·48(0·74 -2·93) | 0·267 |
| 20-24 | 1(Ref) |  | 1(Ref) |  |
| 25-29 | 1·49(0·81 -2·74) | 0·201 | 1·50(0·82 -2·77) | 0·192 |
| 30-34 | 1·54(0·76 -3·11) | 0·228 | 1·58(0·78 -3·19) | 0·206 |
| 35+ | 1·92(0·88 -4·14) | 0·097 | 1·93(0·89 -4·21) | 0·097 |
| **Parity** |  |  |  |  |
| 1 | 1·29(0·73 -2·29) | 0·378 | 1·30(0·73 -2·30) | 0·373 |
| 2-4 | 1(Ref) |  | 1(Ref) |  |
| 5+ | 0·68(0·36 -1·28) | 0·229 | 0·68(0·36 -1·29) | 0·242 |
| **Contraception season** |  |  |  |  |
| Dry | 1(Ref) |  | 1(Ref) |  |
| Wet | 0·78(0·52 -1·17) | 0·234 | 0·85(0·56 -1·29) | 0·439 |
| **HIV status** |  |  |  |  |
| No | 1(Ref) |  | 1(Ref) |  |
| Yes | 0·50(0·19 -1·32) | 0·162 | 0·43(0·16 -1·16) | 0·096 |
| **Sex of baby** |  |  |  |  |
| Male | 1·06(0·71 -1·57) | 0·784 | 1·05(0·71 -1·57) | 0·801 |
| Female | 1(Ref) |  | 1(Ref) |  |
| **American Heart Association category for blood pressure** |  |  |  |  |
| No hypertension | 1(Ref) |  | 1(Ref) |  |
| Stage 1 Hypertension | 1·52(0·80 -2·87) | 0·200 | 1·48(0·78 -2·80) | 0·231 |
| Stage 2 Hypertension | 3·35(1·42 -7·44) | 0·005** | 3·19(1·39 -7·31) | 0·006** |
| **Antepartum Haemorrhage** |  |  |  |  |
| No | 1(Ref) |  | 1(Ref) |  |
| Yes | 3·10(1·77 -5·42) | <0·001** | 2·90(1·65 -5·10) | <0·001** |
| **Tobacco use** |  |  |  |  |
| No | 1(Ref) |  | 1(Ref) |  |
| Yes | 2·10(0·51 -8·69) | 0·307 | 2·19(0·51 -9·35) | 0·292 |
| **Preeclampsia** |  |  |  |  |
| No | 1(Ref) |  | 1(Ref) |  |
| Yes | 1·53(0·83 -2·82) | 0·177 | 1·58(0·84 -2·97) | 0·153 |
| **Mode of delivery** |  |  |  |  |
| Unassisted vaginal/Cephalic | 1(Ref) |  | 1(Ref) |  |
| Operative vaginal | 0·95(0·29 -3·10) | 0·937 | 0·80(0·24 -2·64) | 0·717 |
| Vaginal breech | 1·89(0·79 -4·54) | 0·152 | 1·62(0·66 -3·96) | 0·289 |
| Caesarean section | 1·74(1·01 -3·01) | 0·046 | 1·75(1·01 -3·04) | 0·046 |
| **Birth plurality** |  |  |  |  |
| Singleton | 1(Ref) |  | 1(Ref) |  |
| Multiple | 1·49(0·73 -3·07) | 0·277 | 1·60(0·77 -3·33) | 0·211 |
| **Size for gestational age** |  |  |  |  |
| Small for Gestational Age (<10th centile) | 2·05(1·34 -3·12) | 0·001** | 2·06(1·35 -3·14) | 0·001** |
| Appropriate for Gestational Age (10th to 90th centile) | 1(Ref) |  | 1(Ref) |  |
| Large for Gestational Age (>90th centile) | 0·47(0·15 -1·52) | 0·209 | 0·49(0·15 -1·57) | 0·231 |
| **Country** |  |  |  |  |
| Kenya | — |  | 1(Ref) |  |
| Mozambique | — |  | 1·68(0·95 -2·96) | 0·075 |
| The Gambia | — |  | 1·32(0·63 -2·78) | 0·468 |
| *Akaike’s information criterion (AIC)* | **942·17** | | **943·03** | |
| *Bayesian information criterion (BIC)* | **1136·48** | | **1150·29** | |

BMI = Body Mass Index, PHC = primary health centre. ** p<0·05. *a* Includes women with normal or elevated blood pressure.

*The primary model is Model 3 (adjusted for distal, intermediate, and proximal factors) as reported in Table 3. The sensitivity model adds country (Kenya as reference) as a fixed effect. Complete-case analytical sample: Kenya (2,148 livebirths, 32 stillbirths), Mozambique (1,708 livebirths, 44 stillbirths), The Gambia (846 livebirths, 25 stillbirths).*

# S6 Association between stillbirth and gestational age at birth (complete case analysis)

Consistent with previous studies, there was a strong association between lower gestational age and increased risk of stillbirth on the bivariate analysis.^1-4^ In comparison to delivery at term, delivery at 20^+0^-27^+6^ weeks was associated with a 43-fold increased risk of stillbirth (RR 42·67, 95% CI 28·19 – 64·59) and at 28^+0^ – 31^+6^ weeks with a 17-fold increased risk (RR 16·7, 95%CI 10·86 – 25·68). The risk remained elevated amongst moderate and late preterm births, with birth at 32^+0^-36^+6^ weeks associated with 3·7 times the risk of stillbirth (95%CI 2·53 – 5·40). In contrast to other studies, despite 7·8% of pregnancies delivering beyond 42^+0^ weeks there was no difference detected in stillbirth risk for post-term pregnancies (RR 0·93, 95% CI 0·40-2·16).^5^

In the unselected pregnancy cohort, the median gestational age at recruitment and delivery was 20·7 (IQR: 16·1, 25·3) weeks and 39·3 (IQR: 37·7, 40·7) weeks for women with livebirths, and 18·1 (IQR: 13·4, 22·6) weeks and 35·4 (IQR: 29·4, 38·7) weeks for women with stillbirths. In the selected pregnancy cohort, the median gestational age at recruitment and delivery was 36·9 (IQR: 33·4, 39·0) weeks and 38·3 (IQR: 36·0, 39·1) weeks for women with livebirths, and 35·1 (IQR: 24·3, 38·0) weeks and 35·1 (IQR: 28·6, 38·0) weeks for women with stillbirths.

**References:**

1.	Lawn JE, Ohuma EO, Bradley E, et al. Small babies, big risks: global estimates of prevalence and mortality for vulnerable newborns to accelerate change and improve counting. *Lancet* 2023; **401**(10389): 1707-19.

2.	Okwaraji YB, Suárez-Idueta L, Ohuma EO, et al. Stillbirths: Contribution of preterm birth and size-for-gestational age for 125·4 million total births from nationwide records in 13 countries, 2000-2020. *Bjog* 2023.

3.	Okwaraji YB, Suárez-Idueta L, Ohuma EO, et al. Stillbirth risk by fetal size among 126·5 million births in 15 countries from 2000 to 2020: A fetuses-at-risk approach. *Bjog* 2024.

4.	Lavin T, Pattinson RC, Nedkoff L, Gebhardt S, Preen DB. Stillbirth risk across pregnancy by size for gestational age in Western Cape Province, South Africa: Application of the fetuses-at-risk approach using perinatal audit data. *S Afr Med J* 2019; **109**(12): 927-33.

5.	Management of Full-Term Nulliparous Individuals Without a Medical Indication for Delivery: ACOG Clinical Practice Update. *Obstet Gynecol* 2025; **145**(1): e45-e50.

1. Napolitano, R., et al., *Pregnancy dating by foetal crown–rump length: a systematic review of charts.* BJOG: An International Journal of Obstetrics & Gynaecology, 2014. 121(5): p. 556-565
2. Papageorghiou, A.T., et al., *International standards for foetal growth based on serial ultrasound measurements: the Foetal Growth Longitudinal Study of the INTERGROWTH-21 Project.* The Lancet, 2014. 384(9946): p. 869-879.
3. Self, A., et al., *Second and third trimester estimation of gestational age using ultrasound or maternal symphysis-fundal height measurements: A systematic review.* BJOG: An International Journal of Obstetrics & Gynaecology, 2022. 129(9): p. 1447-1458.
4. World Health Organization, United Nations Children's Fund. The WHO/UNICEF joint monitoring programme estimates on WASH. 2023 [Available from: <https://washdata.org/data>
5. Unger T, Borghi C, Charchar F, et al. 2020 International Society of Hypertension global hypertension practice guidelines. *J Hypertens* 2020; **38**(6): 982-1004.
6. Papageorghiou, A.T., et al., *International standards for fetal growth based on serial ultrasound measurements: the Fetal Growth Longitudinal Study of the INTERGROWTH-21 Project.* The Lancet, 2014. **384**(9946): p. 869-879.
